# Flexible Cation Exchange Environment via Ligand-Free Metal Chalcogenide Thin Films

**DOI:** 10.1021/acsnanoscienceau.4c00023

**Published:** 2024-11-08

**Authors:** Hannah R. Lacey, Kevin D. Dobson, Emil A. Hernández- Pagán

**Affiliations:** † Department of Chemistry and Biochemistry, University of Delaware, Newark, Delaware 19716, United States; ‡ Institute of Energy and Conversion, University of Delaware, Newark, Delaware 19716, United States

**Keywords:** cation exchange, chalcogenides, transition metals, thin films, ligands, HSAB

## Abstract

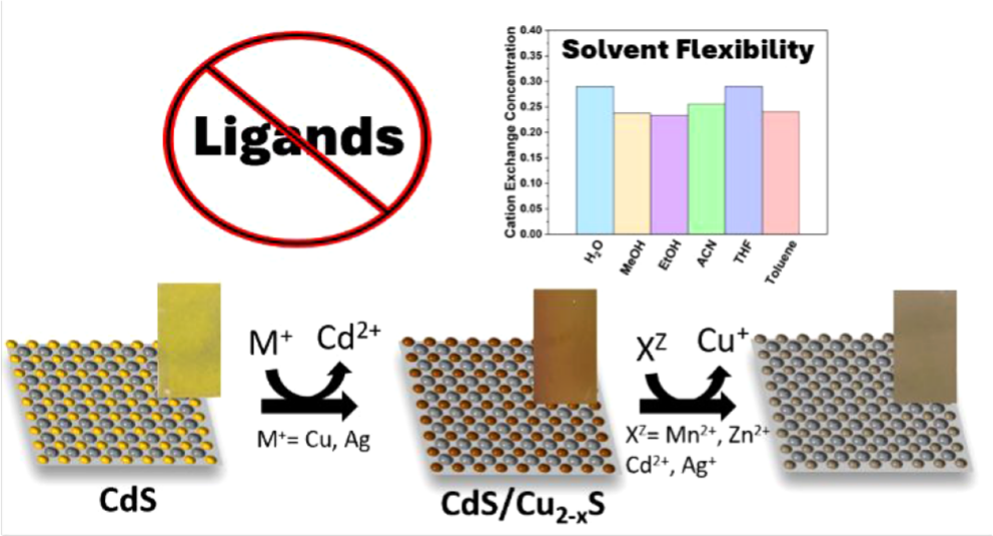

Cation exchange (CE) has emerged as a premier postsynthetic method to carefully tune the chemical composition and properties of nanocrystals with excellent morphology retention. However, reaction conditions are typically dictated by the ubiquitous ligands bound to their surface, limiting their solubility and influencing the thermodynamics/kinetics of the reaction. To bypass these challenges, we report on CE reactions with Cu^+^, Ag^+^, Cu^2+^, Cd^2+^, Zn^2+^, and Mn^2+^ utilizing ligand-free CdS and Cu_*x*_Se_*y*_ thin films as host templates. The exchange reactions could be performed sequentially or simultaneously (i.e., two guest cations) to access compositionally diverse products. The incorporation of cations on the host films was confirmed using SEM-EDS, XPS, and ICP-MS analyses, as well as tracking wavelength shifts in the UV–vis absorption spectra. The flexibility of this approach was demonstrated as reactions were carried out using an array of different guest precursor salts and solvents with a range of polarities. Moreover, the reactions were generalizable among selenide and sulfide films and proceeded under milder conditions in comparison with reported nanocrystal reactions. A ligand-free environment with flexible reaction conditions, as the work herein, could aid in deconvoluting the different factors involved in CE reactions and further expand its use for fundamental research and applications like photovoltaics, optoelectronics, and catalysis.

Immense research in recent decades has been oriented at broadening the repertoire of synthesized nanomaterials to contribute to the expansion of applications such as photovoltaics, photocatalysis, and electronics.^1−9^ Within this repository, cation exchange (CE) has become a prominent technique to postsynthetically alter the chemical composition of colloidal nanomaterials. In this method, cations in solution partially or completely replace cations from a host nanomaterial while retaining their original morphology. The primary principle used to predict and rationalize the feasibility for CE has been hard–soft acid–base (HSAB) theory.^10,11^ For instance, in the CE reaction with CdSe host nanocrystals (NCs), the harder Cd^2+^ cations can be exchanged with a softer Lewis acid, e.g., Ag^+^ using methanol as a hard Lewis base.^12^ In contrast, the reverse reaction (Ag_2_Se + Cd^2+^ → CdSe + 2Ag^+^) typically requires the presence of a soft base, like trioctylphosphine.^13,14^

Metal chalcogenides are commonly used as CE templates as they are earth-abundant and have tunable compositions, controllable morphologies, and properties suitable for a breadth of applications.^10−12,15−20^ There have been several reports of complex nanoheterostructures (NHS) fabricated postsynthetically with partial CE by tuning the concentration of precursors, reaction time, and temperature.^16,21−24^ NHSs have received extraordinary interest in the literature as they possess the ability to integrate properties of two or more materials, influence charge separation and recombination, and may promote synergy between interfaces.^21,25−28^ The Schaak group showed that partial CE could be utilized as a scalable and generalizable tool to synthesize metal sulfide nanorods with up to six materials, eight segments, and 11 internal interfaces employing roxbyite copper sulfide nanorods.^23,24^

While CE in colloidal nanocrystals has proven to be a powerful postsynthetic technique enabling the careful tuning of composition/structure, ubiquitous ligands bound to the nanocrystal surface dictate the reaction conditions. For instance, exchange reactions with host NCs synthesized with the common ligands oleylamine and/or oleic acid are carried out in nonpolar solvents to prevent agglomeration.^16,29^ Nonpolar solvents, such as octadecene and dioctyl ether, tend to be inert and provide a wide temperature window. However, these benefits come at the expense of lower solubility of the guest precursor salt and, thus, set constraints for the reaction. For example, reaction conditions may require the use of bulky anions (e.g., triflate), complexation with oleylamine/oleic acid, or the use of higher temperatures, even when not desired. This limitation in solubility can be bypassed by running the CE in polar solvents, in which case the NCs are required to have hydrophilic ligands. This is possible by one of two routes: ligand exchange where original hydrophobic ligands are replaced with hydrophilic ones or synthesis of host NCs in polar conditions with hydrophilic ligands. However, both options have limitations as ligand exchange usually creates surface defects that can negatively impact the properties of the NCs,^30^ and with exception of metal NCs, syntheses in polar environments tend to offer less control over the size and shape of the host NCs.^31,32^

Furthermore, the differences in ligand and solvent environments make it challenging to draw comparisons among the CE reactions. Ligands on host NC can hinder the reaction kinetics/thermodynamics, impact the site where CE is initiated, and dictate the phase of the CE product. The Rioux group demonstrated that different capping agents had varying enthalpies in the CE reaction between CdSe and Ag^+^, impacting the reaction rate.^33^ Similarly, Shim and coworkers found that linear ligands provided a greater barrier on the partial CE between Cu_2-x_S nanorods and Ga^3+^ in comparison to bulky ligands, as the linear ligands can pack more densely on the surface hindering CE.^22^ Moreover, the Macdonald group found that the degree of electron donating ability in phosphine ligands dictated whether the thermodynamic or metastable phase was formed in the CE of hexagonal Cu_2_S to CuFeS_2_.^34^ Hence, it would be beneficial to have a simplified system to more closely investigate the individual components of the exchange.

We posit that ligand-free thin films allow for greater flexibility in terms of selection of precursor salt, solvent, and extracting base. This flexibility could enable fundamental comparison experiments under a range of conditions that can aid in the creation of design principles. Moreover, thin films are more compatible with device fabrication infrastructure, which provides an easier path to translate the fundamental research toward applications. While thin films have not been extensively studied as templates for cation exchange, there have been a few encouraging literature reports on single-crystal cation exchange. In the 1970s, several groups reported on the use of CE to modify the surface of single crystals resulting in the formation of semiconductor heterointerfaces.^35−40^ More recent reports are limited, but the focus has been on improving the properties of thin films via CE. For instance, Snaith and coworkers employed CE to tune the bandgap of lead iodide perovskite (APbI_3_) thin films by exchanging the “A” site cation. Similarly, Sahu et al. reported on the CE of Cu_2-x_Se thin films with Ag^+^ to improve the thermoelectric properties.^41,42^ Analogous to thin films without a substrate, CE on colloidal 2D materials has also been reported as illustrated by the Huang group with the CE between CuTe nanosheets and various noble metals.^20^ Despite these reports, there remains a gap in the translation of knowledge from NC cation exchange to thin films, providing us an opportunity to expand on this postsynthetic method.

Herein, we demonstrate the versatility and flexibility of CE for thin films starting from ligand-free cadmium sulfide and copper selenide host films. These materials are routinely used in the nanocrystal CE literature, providing a foundation for us to build upon. Polycrystalline cadmium sulfide thin films were fabricated in-house using chemical bath deposition (CBD), while copper selenide films were fabricated via a thermal coevaporation technique. Cation exchanges were successfully performed with cations including Cu^+^, Ag^+^, Cu^2+^, Cd^2+^, Zn^2+^, and Mn^2+^. Successive reactions and one-pot reactions involving multiple cations were employed to increase the compositional complexity of the host material. The ligand-free environment enabled the use of various salts and solvents with a range of counterions and polarities.

## Results and Discussion

### Host Thin Films

**Figure 1:**
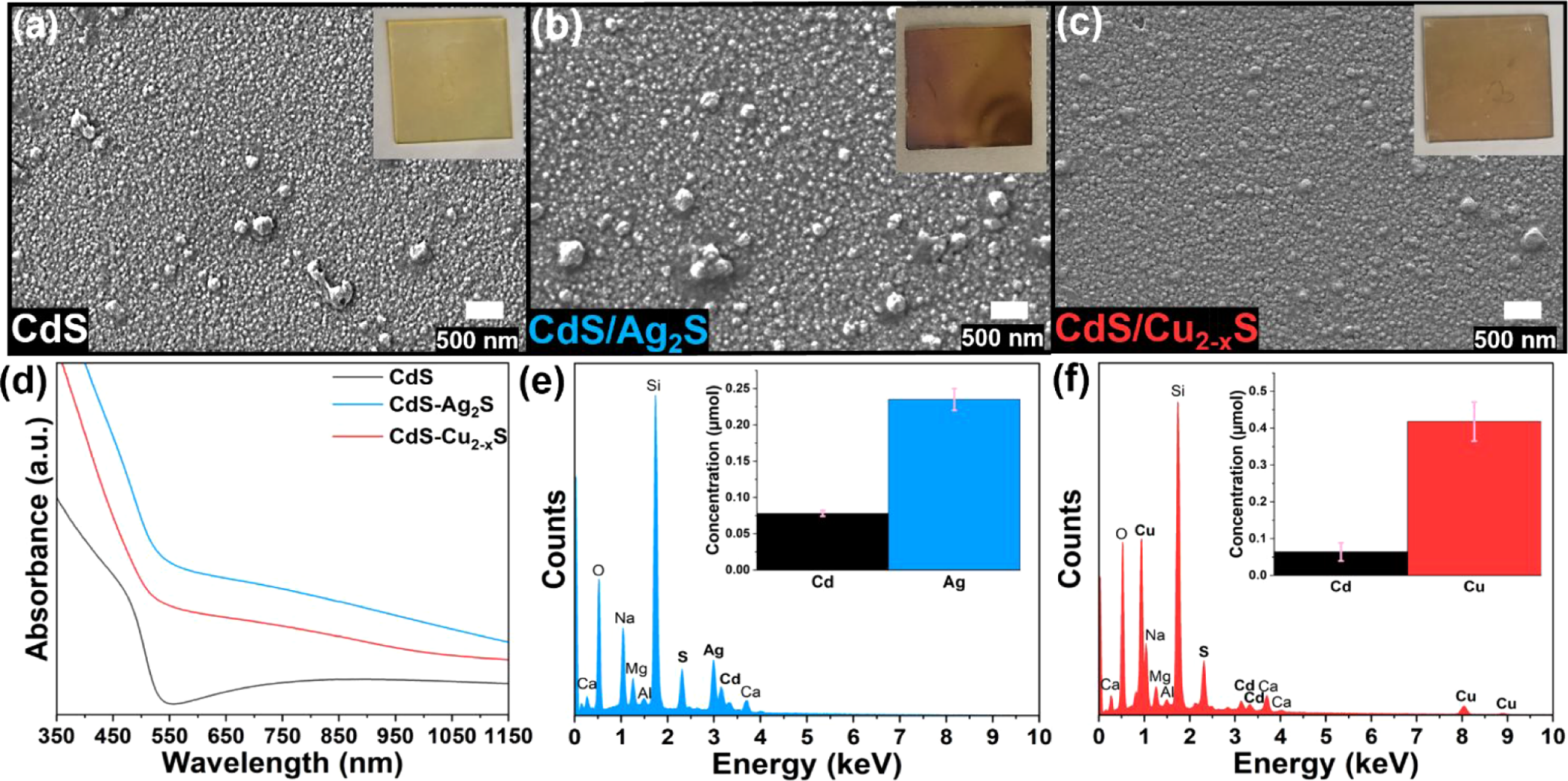
SEM images showing the morphology of (a) the pristine CdS host film and (b) CdS postexchange with Ag^+^ and (c) Cu^+^. Insets in the right corner are pictures of each respective film. (d) UV–vis absorbance spectra of the first-generation exchanges with respect to CdS. EDS spectra with bar graph insets corresponding to the cation concentrations obtained from ICP-MS analysis of the films after exchange with (e) Ag^+^ and (f) Cu^+^. The cation concentrations with error bars representative of the standard error of the mean were obtained from the averaging of three and eight trials for the Ag and Cu cation exchanges, respectively. Au and Pd present in the EDS spectra were due to sputtering used to avoid charging during imaging, and the other elements (i.e., Si, Ca, O, Na, Mg, and K) are present from the microscope slide.

Metal chalcogenides were chosen as model systems because they are commonly used as templates in the CE of nanocrystals due to their high cation mobility, flexibility, and tunability.^21,29^ This selection also allowed us to build upon existing knowledge and expand into the thin film system. In particular, we selected CdS and Cu_*x*_Se_*y*_ to use as host materials in the CE. CdS thin films can be fabricated using established CBD techniques, which are cost-effective and do not require any specialized equipment. Based on modified procedures,^43,44^ we were able to obtain consistent CdS films with an approximate thickness of 70 nm. Visually, the films exhibit the yellow color characteristic of bulk CdS, and the absorption spectrum showed an onset of absorption around 545 nm (Figures 1a inset and S1a), consistent with literature reports.^45,46^ Scanning electron microscopy (SEM) analysis revealed that the films were polycrystalline (Figures 1a and S1a), and atomic force microscopy (AFM) revealed an average roughness mean squared (*R*_ms_) of 12.2 ± 1.1 nm (Figure S2 and Table S1). Structural characterization by grazing incidence X-ray diffraction (GIXRD) indicated that films are predominantly zincblende CdS with minor reflections correlating to the wurtzite phase and a calculated average Scherer crystallite size of 5.7 nm (Figure S3). Figure S4 shows a representative Raman spectrum of a film with active modes at 300 and 600 cm^–1^, consistent with the reported 1LO and 2LO vibrations of CdS.^47,48^ A bulk, semiquantitative composition of 1:1 Cd:S was obtained by energy-dispersive spectroscopy (EDS) similar to that obtained via X-ray photoelectron spectroscopy (XPS) which is more representative of the surface composition (Figures S1b, S5 and Table S2). The cadmium concentration was determined with inductively coupled plasma mass spectroscopy (ICP-MS) (Table S3) as a baseline for comparison with postexchanged samples. To ensure that the results were not system-dependent, we employed copper selenide thin films fabricated via thermal evaporation. A different fabrication technique allowed us to evaluate the dependence, if any, of the exchange on surface morphology (size, shape, and packing of grains). The Cu_*x*_Se_*y*_ films were also polycrystalline (Figure S1c inset) with an average *R*_ms_ value of 68.5 ± 0.6 nm (Figure S6 and Table S4). The reflections of the GIXRD pattern suggest that the films are a mixture of the orthorhombic and cubic phases (Figure S7). The UV–vis spectra of these films shows increased absorption in the NIR region indicative of a localized surface plasmon band (Figure S1c).^8,9,29,49^ The bulk composition of the films was determined with SEM-EDS (Figure S1d) and ICP-MS (Figure S1d inset and Table S5). ICP-MS analysis revealed that these host films were copper-rich (Cu:Se ratio of 2.4). In contrast, XPS quantification suggests a substoichiometric composition with a Cu:Se of 1.7 with copper in +1 and +2 oxidation states as evidenced by the satellite peaks in the Cu 2p spectrum (Figure S8 and Table S6).^50,51^ Raman spectroscopy measurements were also performed, but the films proved not to be Raman active (Figure S9), perhaps due to the mixed phase nature of the samples.

### First-Generation CE Reactions with CdS and Cu_*x*_Se_*y*_

All CE reactions discussed herein were performed with a large excess of guest cation precursors to create a concentration gradient used to drive the flux of incoming cations and promote the exchange. For all CE reactions with the CdS host film, the reaction time was maintained at an hour for consistency purposes, based on time studies performed with Ag^+^ (Table S7). We first set out to test the CE reaction capabilities of the CdS films by starting with an Ag^+^ exchange adapted from the NCs literature.^12,52^ In brief, a CdS film was introduced to a solution of AgNO_3_ in acetonitrile at room temperature and allowed to react for 1 h. As the reaction proceeded, we observed that the film color gradually changed from yellow to dark brown, indicating the replacement of Cd^2+^ for Ag^+^ (Figure 1a,b insets). Figure 1b shows an SEM image of the film postexchange revealing no evidence of film degradation throughout the reaction. This stability was validated through AFM (Figure S10, Table S1) as the surface topography *R*_ms_ value of 14.4 ± 2.9 nm was similar to that for the pristine films (*R*_ms_ = 12.4 ± 1.1 nm). The Raman spectrum was consistent with that of CdS, indicating partial exchange (Figure S11). EDS analysis (Figure 1e) showed the appearance of the Ag L_α_ peak and a decrease in intensity of the Cd L_α_ signal supporting the exchange of the cations, which was further validated through ICP-MS and XPS analyses (Figures 1e and S12, Tables S8 and S9). The Ag 3d XPS spectrum (Figure S12) displayed binding energies for the Ag 3d_5/2_ and 3d_3/2_ peaks of 368.6 and 374.6 eV, as expected for Ag^+^.^50,53^ No notable difference was observed in the S 2p spectrum (Figures S5 and S12). The GIXRD pattern shows reflections for CdS (in agreement with the Raman spectrum) and the emergence of new reflections. While the broadness and low intensity of the new reflections make unambiguous phase identification difficult, there is some resemblance to the reference pattern of acanthite Ag_2_S (Figure S13). The crystallite size was found to be 6.3 nm based on Scherer analysis, which is close to the value calculated for the host material. The resulting films showed a red-shifted absorbance relative to that of the CdS host film (Figure 1d). This shift was expected based on the reported bandgaps for CdS and Ag_2_S of 2.4 and 1.0 eV, respectively. To further assess this proof of concept, we probed the CE with Cu^+^ following modified literature procedures.^54^ CdS films were exposed to a methanolic solution of [Cu(CH_3_CN)_4_]PF_6_ leading to a color change in the film from yellow to brown (Figure 1a,c insets). The SEM image in Figure 1c shows that the morphology was maintained throughout the reaction, while the AFM image showed no discernible change in topography (*R*_ms_ 10.3 ± 5.2 nm) (Figure S14, Table S1). The presence of Cu and decrease of Cd were evident in both the EDS and ICP-MS analysis (Figure 1f, Table S10). XPS analysis also showed the incorporation of Cu into the film upon CE, with binding energies for the Cu 2p_3/2_ and 2p_1/2_ peaks of 932.3 and 952.2 eV, and no satellite peaks, as expected for Cu^+^ (Figure S15).^50,51^ No notable difference was observed in the S 2p spectrum (Figure S5 and S15). Interestingly, the XPS measurements (Table S11) suggest a Cd-rich surface composition, in stark contrast to the bulk composition from ICP-MS and EDS analyses. We further investigated this result with XPS depth profiling (Figure S16). The profile shows that the copper concentration increases as deeper depths are probed, while the cadmium concentration decreases, overall correlating with the bulk measurements. This result suggests that the Cu cation exchange may follow a different diffusion pathway than the Ag cation exchange. Differences between the reaction path have also been reported for cation exchange of CdS nanorods with these two cations.^54,55^ The absorption spectrum of the exchanged product showed a red shift (Figure 1d) in accordance with the smaller bandgap for copper sulfide in comparison to cadmium sulfide. The GIXRD pattern showed the disappearance of the CdS reflections and appearance of one broad reflection at 46.7°, which potentially matches the 220 plane of digenite copper sulfide and gives a Scherer crystallite size of 4.8 nm (Figure S17). The Raman spectra did not show any of the modes associated with CdS, which is in agreement with the diffraction pattern (Figure S18). Also missing was the S–S mode reported in the literature and observed here for other samples (*vide infra*). This exchange could also be performed with an aqueous solution of Cu(OAc)_2_ as demonstrated in Figure S19. When comparing the ICP-MS and EDS results of the CE with [Cu(CH_3_CN)_4_]PF_6_ vs Cu(OAc)_2_, greater exchange (i.e., higher Cu concentration) was obtained when using [Cu(CH_3_CN)_4_]PF_6_ (Figures 1f and S19, Tables S10 and S12). However, XPS analysis of the samples exchanged with Cu(OAc)_2_ shows more copper than cadmium, opposite to [Cu(CH_3_CN)_4_]PF_6_ (Figure S20, Table S13). Our initial rationale was that Cu^+^ has a higher mobility and is a softer Lewis acid (absolute hardness, η = 6.28) in comparison to Cu^2+^(η = 10.88).^11,56^ However, the Cu 2p spectrum of the samples exchanged with Cu(OAc)_2_ matched that expected for Cu^+^ with the notable absence of the characteristic satellite peaks seen for Cu^2+^ (Figure S20). This result was quite surprising, as, besides sulfur, there is no obvious reducing agent. While the S 2p and the Raman spectra show some indication of oxidized S species (Figures S20 and S21), it is difficult to determine their origin given that the reaction is carried out in water. Nevertheless, it is possible that this redox process influences the degree of exchange and the surface composition differences. The chelating nature of the acetate ligand can result in a stronger binding to the metal cation,^57^ which can also contribute. From here on, the reactions between CdS with Ag^+^ and Cu^+^ are categorized as first-generation cation exchange and will be referred to as CdS/Ag_2_S and CdS/Cu_2-x_S, respectively.

**Figure 2:**
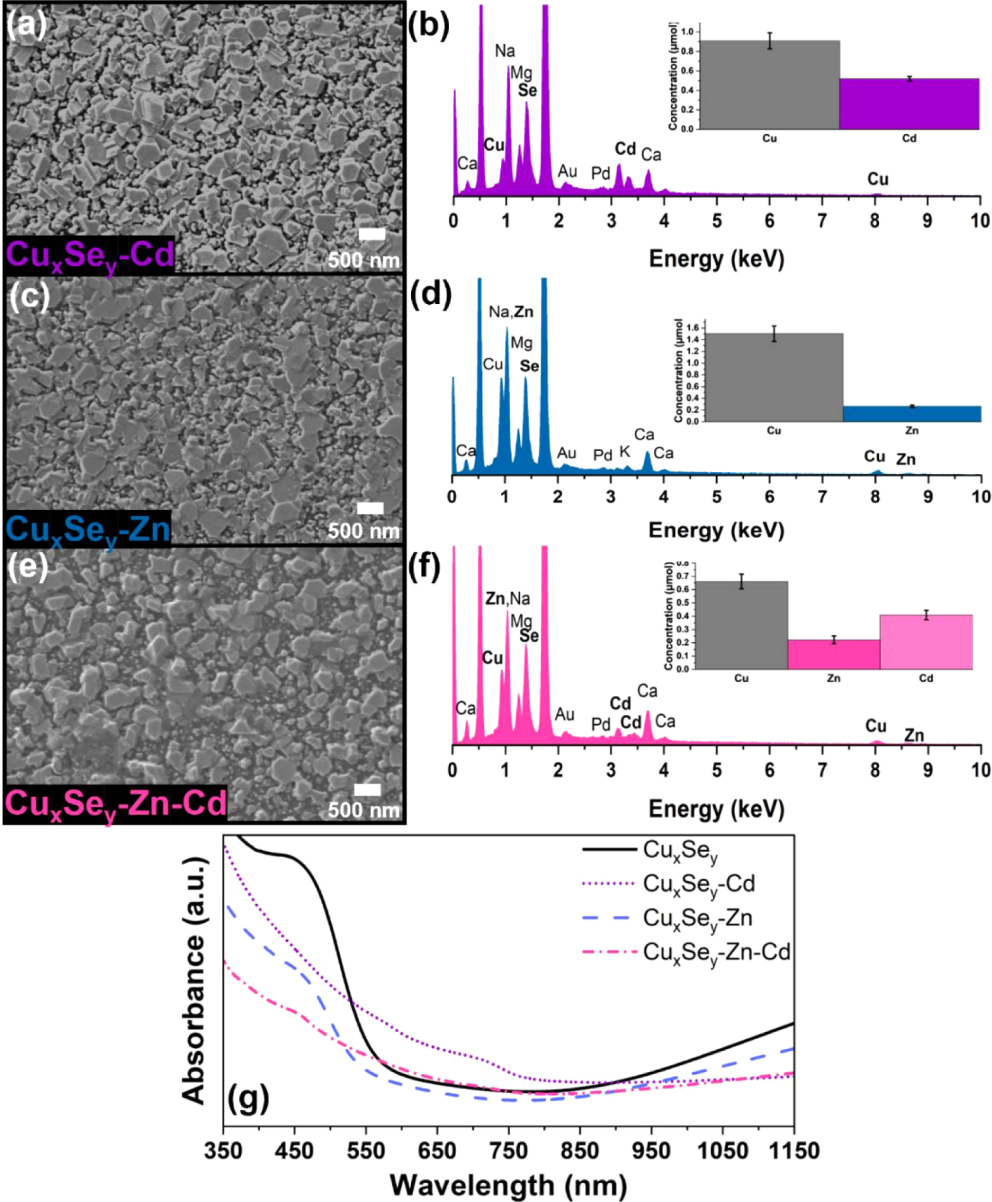
First-generation exchanges with the Cu_*x*_Se_*y*_ host. SEM images representing the morphology of (a) Cu_*x*_Se_*y*_ postexchange, with Cd^2+^, (c) Zn^2+^, and (e) sequential exchange with Zn^2+^ and Cd^2+^, respectively. EDS spectra with bar graph insets corresponding to the cation concentrations obtained from ICP-MS analysis of the films after exchange with (b) Cd^2+^, (c) Zn^2+^, and (e) sequential exchange Zn^2+^ and Cd^2+^. (g) UV–vis absorbance spectra of the Cu_*x*_Se_*y*_ host film after exchange with Cd^2+^, Zn^2+^, and the sequential exchange with Zn^2+^ and Cd^2+^.

To expand this methodology to other chalcogenides and ensure that the process was not system-dependent, we evaluated copper selenide thin films as host materials (Figure S1c–f). First, a CE reaction with Cd^2+^ was performed where the reaction was prepared in the glovebox by dissolving CdI_2_ in acetonitrile and transferred to a Schlenk line to be placed under N_2_ flow at 70 °C for 2 h. It is important to ensure this reaction is kept under an inert atmosphere, as air exposure causes the host film to degrade. CdI_2_ was chosen as an exchange precursor in an attempt to drive the solvation energy as I^–^ is a soft Lewis base (η = 3.70),^56^ thus more favorable to bind Cu^+^, a soft Lewis acid. The morphology and topography remained consistent throughout the reaction as evident from SEM and AFM analyses with an *R*_ms_ value of 61.1 ± 0.4 nm (Figures 2a and S22, Table S4). The EDS spectrum (Figure 2b) showed the appearance of Cd with a concomitant decrease in the Cu signal. ICP-MS analysis of the exchanged films was in agreement with the EDS result (Figure 2b, Table S14). The surface composition determined by XPS was close to 1:1 Cd:Se (Table S15). The Cd spectrum (Figure S23) showed 3d_5/2_ and 3d_3/2_ binding energies of 405.2 and 411.9 eV as expected for Cd^2+^.^50,58^ The Se 3d spectrum is consistent with that observed for the pristine films but also shows peaks corresponding to iodide from the precursor despite thorough rinsing after the reaction (Figure S23). In contrast, the Cu 2p spectrum (Figure S23) for these samples is significantly different from that of the pristine films. The satellite peaks associated with Cu^2+^ are absent, and the binding energies for the Cu 2p_3/2_ and 2p_1/2_ peaks of 932.5 and 952.3 eV match those of Cu^+^.^50,51^ This result suggests that Cu^2+^ observed on the pristine films is primarily at the surface and gets depleted during exchange. The GIXRD pattern of the exchanged films revealed reflections that matched the reference pattern for zincblende CdSe (Figure S24). Likewise, the Raman spectrum displayed active modes at 203 cm^–1^, 260 cm^–1^, and 404 cm^–1^ which have been reported as the 1LO mode of zincblende CdSe, Se–Se bond vibration, and 2LO of CdSe, respectively (Figure S9).^59,60^ Successful incorporation of Cd^2+^ (even if partial) would lead to a red shift of the absorbance given that CdSe has a narrower band gap than Cu_*x*_Se_*y*_, regardless of the stoichiometry. This expected shift was confirmed via absorption spectroscopy (Figures 2g and S25a). A CE reaction between Cu_*x*_Se_*y*_ and Zn^2+^ was performed under identical conditions, except that ZnBr_2_ was used as the guest precursor salt. The absorption spectrum of the exchanged product (Figures 2g and S25b) did not show any significant changes, which can be anticipated as both copper and zinc selenide have similar wide bandgaps. Similar to the reaction with Cd, evidence for zincblende ZnSe emerged from the GIXRD pattern (Figure S26). SEM (Figure 2c) and AFM images (Figure S27, Table S4) before and after the reaction showed little discrepancy (*R*_ms_ of 67.4 ± 5.6 nm), and the presence of Zn was confirmed by EDS, ICP-MS analysis, and XPS (Figures 2d and S28, Table S16 and S17). Here too, the surface composition determined by XPS was nominally 1:1 Zn:Se (Table S17). The Zn spectrum (Figure S28) was consistent with Zn^2+^ with an fwhm value of 1.8 eV for the 2p_3/2_ peak.^50,61^Figure S28 also displays the Se 3d and Cu 2p spectra. The Se spectrum was consistent with that observed for the pristine films, while the Cu 2p spectrum only showed the presence of Cu^+^ similar to that observed in the Cd exchange.

In comparing these two exchanges, similar surface compositions were extracted from the XPS measurements (Table S15, S17). However, the ICP-MS data revealed that the Cd^2+^ exchange resulted in significantly more uptake than the CE with Zn^2+^ despite both being Lewis acids of similar hardness (η, 10.29 Cd^2+^ vs 10.88 Zn^2+^)^56^ (Figure 2b,d insets, Tables S14 and S16). Following literature reports,^19,25,62^ calculations of the reaction’s Δ*G*° based on constants taken from the CRC handbook^63^ suggest the exchange with Cd^2+^ (−7.9 kJ/mol) is slightly more thermodynamically favorable than exchange with Zn^2+^ (−4.6 kJ/mol). This favorability was also observed when Cd^2+^ and Zn^2+^ were both introduced to the host film in an effort to increase the complexity of the exchange product. At 1:1 and even 1:2 Cd^2+^: Zn^2+^ ratio, the exchange with Cd^2+^ completely dominated with negligible incorporation of Zn^2+^(Figure S29). To bypass this limitation, we instead performed a sequential reaction where ZnBr_2_ dissolved in acetonitrile was first introduced to the film and allowed to react for 30 min. Subsequently, CdI_2_ dissolved in acetonitrile was injected into the reaction flask and allowed to react for another 30 min. The reaction was maintained at 70 °C under a N_2_ flow. The resulting film showed a slight red shift in the absorption spectrum (Figure 2g and S3c) as expected based on Cd^2+^ incorporation. SEM imaging before and after the exchange showed that the films remained invariable (Figure 2e). The presence of both Zn and Cd was confirmed by EDS and ICP-MS analyses (Figure 2f, Table S18). XPS analysis also showed the presence of both Zn^2+^ and Cd^2+^ (Figure S30, Table S19). However, in contrast to the bulk measurements performed, zinc was more abundant at the surface. A possible explanation for this observation is that zinc was introduced to the film first and thus exchanged with more copper ions at the surface of the material, so the cadmium ions introduced later in the reaction exchange with the copper ions further in the bulk of the film.

One distinct difference between the thin film model systems utilized here and the exchange using NCs is the longer reaction times. The thin films have a 2D geometry with an area of ∼2.5 cm^2^ and ∼70 nm thick. On the other hand, the NCs in the referenced CE literature are typically <30 nm in diameter and freely dispersed in solution. Thus, based on these differences, the longer reaction time and only partial CE can be rationalized based on diffusion length.^10^ It is important to mention, however, that the thin film copper selenide exchange reactions were performed in milder reaction conditions than those reported in the NC literature.^16,29,49^ Another notable difference was observed for the exchanges where the soft Lewis acid Cu^+^ is replaced with harder Lewis acids (i.e., Cd^2+^, Zn^2+^, and Mn^2+^). In this type of reaction, it is commonplace for a soft Lewis base such as trioctylphosphine (or other trialkylphosphines) to be added to the reaction as a driving force to extract the soft host cation.^13,14^ In this thin film system, however, we found that it was not necessary to add phosphines to the reaction mix. These reactions were able to proceed with a halide guest precursor salt and acetonitrile. This suggests that the halide counterion acts as a “soft” base in the reaction to extract the “soft” host cation. A similar observation was reported by Huang et al. on CE transformations of copper chalcogenide nanostructures via a halide precursor salt using ethylene glycol or dimethyl sulfoxide as a solvent to form noble metal chalcogenides.^20^

We posit that this reaction modality is possible because of the increased solubility of the halide salts in acetonitrile, whereas these salts in nonpolar solvents, commonly used in NC CE, are insoluble and hence require high temperature and complexation to dissolve. When phosphine was added, the exchange proceeded with concomitant film degradation. While phosphines are known to etch metal chalcogenides,^64,65^ etching is not commonly reported in NC CE reactions, perhaps due to the presence of surface ligands inhibiting this process.

### CdS-Ag Precursor/Solvent Study

**Figure 3:**
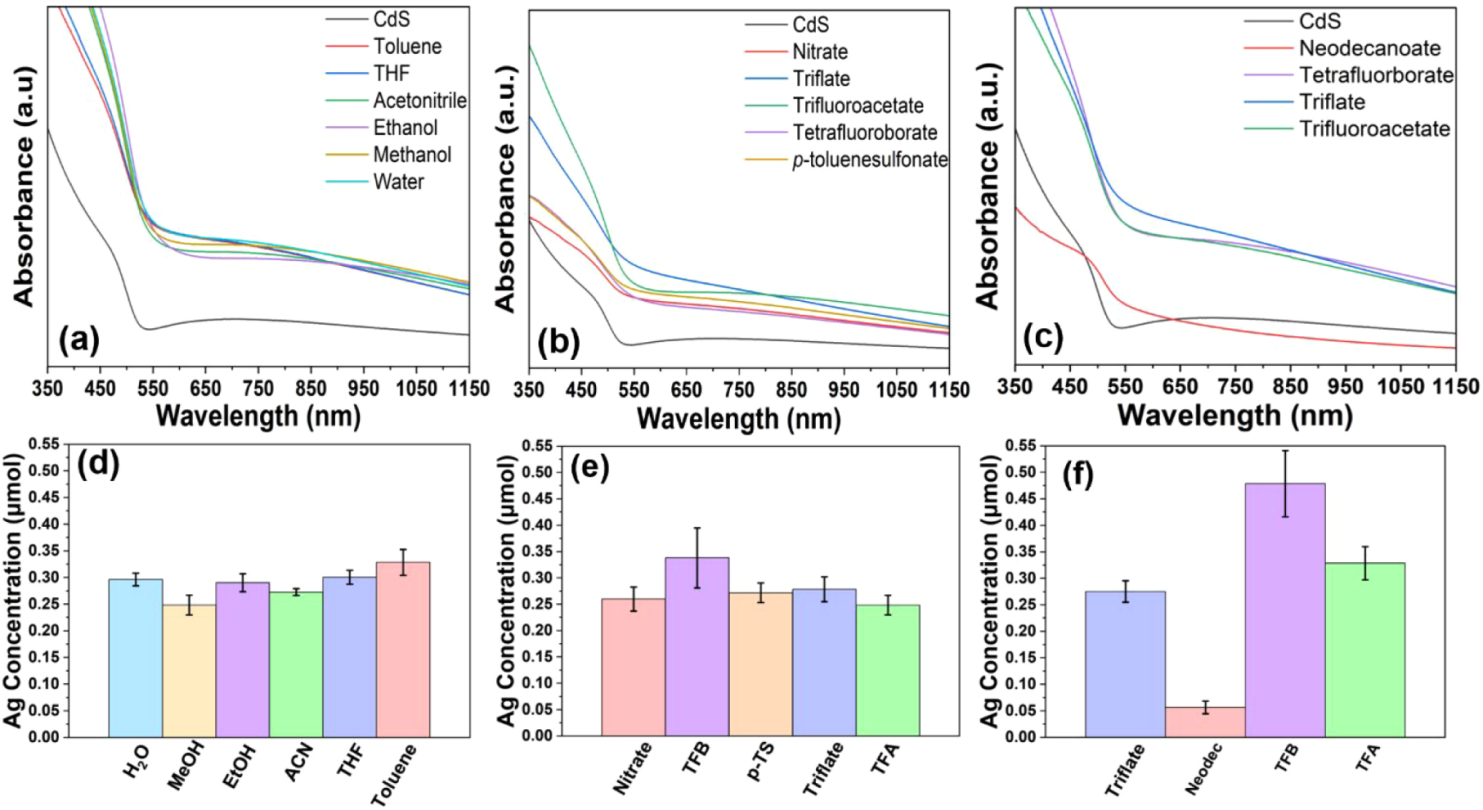
UV–vis spectra of first-generation exchange of CdS with Ag^+^ under various reaction conditions; (a) utilizing numerous solvents with a range of polarities all with silver trifluoroacetate (TFA) as the precursor salt and using various precursor salts with (b) MeOH and (c) toluene as solvent. All reactions proceeded for 1 h at room temperature under continuous stirring except for the reaction with silver neodecanoate in toluene, which proceeded for 3 h at 65 °C. Bar graphs corresponding to the silver concentration postreaction obtained from ICP-MS analysis of (d) different solvents with TFA as the precursor salt and using various precursor salts (TFB = tetrafluoroborate, neodec = neodecanoate) with (e) MeOH and (f) toluene as the solvent. Error bars representative of the standard error of the mean were obtained from the average of four trials.

To further demonstrate the advantage of a ligand-free host material, various precursor salts and solvents with diverse polarities and counterions were utilized in the first-generation exchange between CdS and Ag^+^. We chose this specific reaction for our investigation, as there are numerous silver precursor salts with different counterions with a wide range of solubilities. In all reactions, a host film was introduced to a solution of a silver precursor salt dissolved in various solvents. To evaluate the counterions, silver salts of tetrafluoroborate (TFB), *p*-toluenesulfonate (p-TS), trifluoroacetate (TFA), and nitrate were dissolved in methanol, while neodecanoate (neodec), TFB, triflate, and TFA were dissolved in toluene. To test the different solvent polarities, AgTFA was dissolved in toluene, THF, acetonitrile, ethanol, methanol, and water. In general, all reaction combinations yielded exchanged films as evidenced by the red shift of the UV–vis absorption spectra and similar concentrations of Ag obtained from ICP-MS analysis (Figure 3 and Tables S20–S22), confirming the flexibility allotted by the thin film model system. More specifically, the results suggest that at least for this reaction modality, solvent choice had little to no impact on the degree of CE, regardless of its polarity. Solvents would play a key role when precursors have significantly different solubilities as we saw for the Cu^+^ exchange with halide salts (*vide supra*). When the different counterions used in this reaction were accessed, all combinations yielded a similar result with two exceptions. The reaction with silver neodecanoate in toluene showed significantly less Ag^+^ incorporation based on the UV–vis and ICP-MS results (Figure 3, Tables S20–S22). We posit that this result is a consequence of the chelating effect where the bidentate carboxylates bind silver cations more strongly and the bulky neodecanoate complex diffuses more slowly through the grain boundaries,^57^ both hindering the CE. However, the chelate effect was not observed with the TFA salt which could be attributed to its strong electron withdrawing ability. Second, reactions with Ag-TFB using both MeOH and toluene solvents unexpectedly resulted in a greater uptake of Ag. A possible explanation for this observation is that tetrafluoroborate is the weakest coordinating anion out of all of the ones evaluated.

### Second-Generation Exchanges from CdS Host Films

**Figure 4:**
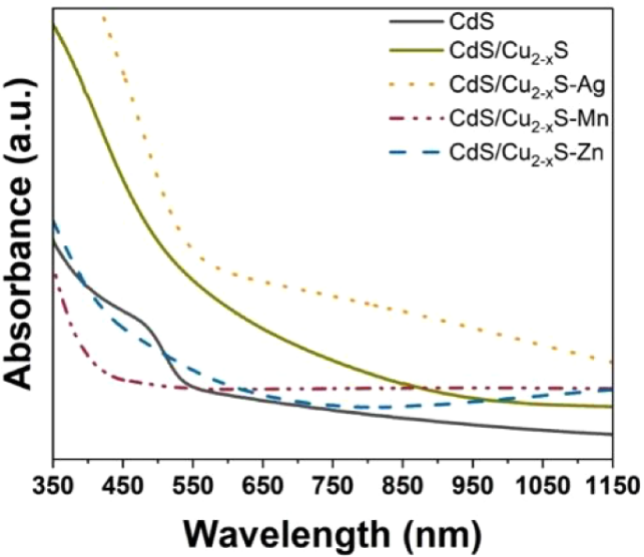
UV–vis absorbance spectra of second-generation exchanges with respect to CdS/Cu_2-x_S and pristine CdS with Ag^+^, Mn^2+^, and Zn^2+^.

**Figure 5:**
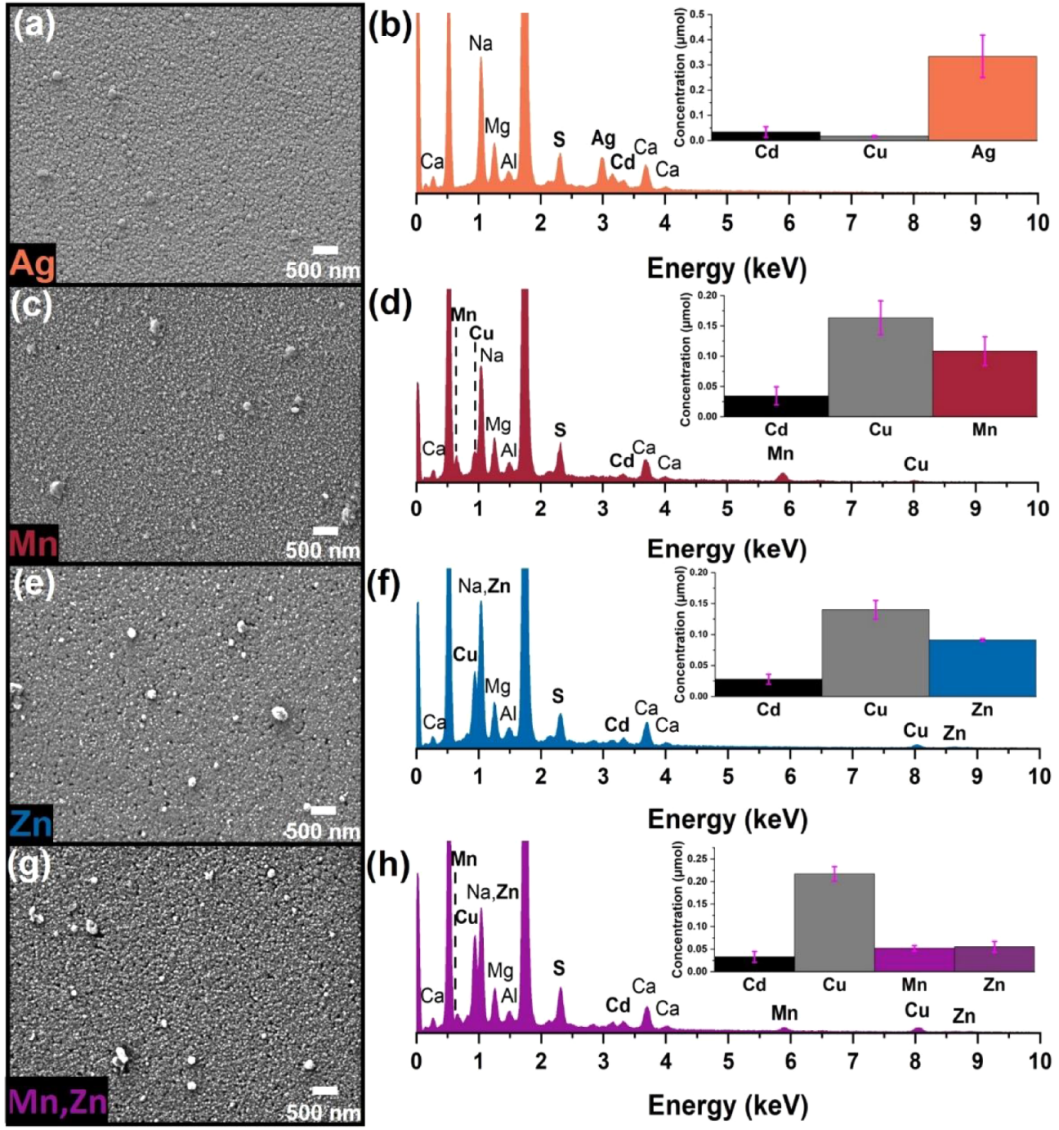
Second-generation exchanges with CdS/Cu_2-x_S as host. SEM images showing the morphology upon exchange with (a) Ag^+^, (c) Mn^2+^, (e) Zn^2+^, and (g) Mn^2+^ and Zn^2+^ simultaneously. EDS spectra with bar graph insets corresponding to the cation concentrations obtained from ICP-MS analysis of the films after exchange with (b) Ag^+^, (d) Mn^2+^, (f) Zn^2+^, and (h) simultaneous exchange with Mn^2+^ and Zn^2+^. The cation concentrations with error bars representative of the standard error of the mean were obtained from the averaging three trials for CE reactions with Ag and Zn, seven for Mn reaction, and six for Mn and Zn reaction.

To determine whether the reactions established for the copper selenide exchanges could be extended to other chalcogenides, second-generation CE reactions were performed on the first-generation template CdS/Cu_2-x_S described above. A second-generation CE was first performed between CdS/Cu_2-x_S and Ag^+^ where the host film was introduced to a solution of AgNO_3_ in acetonitrile at room temperature for 1 min.^42^ This reaction proved to happen readily and resulted in a nearly complete exchange. We immediately observed a color change from light brown to dark brown which resulted in a slight red shift in the UV–vis absorption spectrum (Figures 4 and S31a). This was in line with our expectations as the bandgap of Ag_2_S is slightly narrower than the bandgap of Cu_2-x_S. The morphology and topography of the film before and after the exchange were maintained as evidenced by SEM and AFM imaging, respectively, with an *R*_ms_ value of 11.8 ± 0.6 nm (Figures 5a and S32, Table S1). The chemical composition was confirmed with EDS and ICP-MS (Figure 5b, Table S23). It is worth noting that this reaction also proceeded with the Cu_*x*_Se_*y*_ system under identical conditions (Figure S33). Additionally, an exchange with Mn was performed where the CdS/Cu_2-x_S host film was introduced to a solution of MnI_2_ in acetonitrile on a Schlenk line under N_2_ flow at 70 °C for 1 h using the same air free conditions as outlined for the copper selenide reactions. The resulting thin film product appeared taupe in color and with a blue shift in the UV–vis absorption spectrum (Figures 4 and S31b), correlating with what would be expected based on the bandgap of MnS (∼2.7- 3.7 eV).^66^ SEM imaging (Figure 5c) showed that the thin film morphology was maintained throughout the reaction, and EDS analysis (Figure 5d) showed the appearance of Mn peaks and a decrease of Cu peaks indicating that exchange of cations occurred. The exchange was validated through ICP-MS analysis (Figure 5d, Table S24). Similarly, a CE reaction was performed with Zn^2+^ following the same procedure as the Mn reaction. In both cases, a blue shift would be expected based on the bandgap of ZnS of 3.6 eV. This shift was corroborated by the evolved UV–vis absorption spectra (Figures 4 and S31c). Similarly, the morphology and topography remained consistent with an *R*_ms_ of 11.5 ± 2.3 nm (Figures 5e and S34, Table S1), and the incorporation of guest cations was confirmed with EDS and ICP-MS (Figure 5f, Table S25). The GIXRD patterns for the second-generation exchanges had no discernible peaks, suggesting a loss of crystallinity (Figure S35). The XPS spectra (Figures S36–S38 and Tables S26–S28) of these samples showed Ag^+^, Zn^2+^, and Mn^2+^ for the corresponding reactions. Raman spectroscopy measurements revealed the emergence of a mode around 470 cm^–1^ in each of the samples, which, as mentioned above, correspond to S–S bonds that arise due to surface oxidation^25,67^ (Figure S39).

Moreover, we set out to employ multication reactions to increase the complexity of the films and further modify the optical properties. To achieve this, we combined cation salt solutions based on successful single second-generation exchanges. To test this concept most easily, a one-pot reaction involving both Zn^2+^ and Mn^2+^ was performed on a CdS/Cu_2-x_S host. Here, a solution of ZnBr_2_ and MnI_2_ in acetonitrile was introduced to the host film under a N_2_ environment and allowed to react at 70 °C for 1 h. We observed a blue shift in the UV–vis spectrum (Figures S6 and S31d) as expected based on the wide bandgaps of both ZnS and MnS. The morphology was maintained as evident in the SEM image (Figure 5g). Likewise, the presence of Mn and Zn was confirmed via EDS, ICP-MS, and XPS analyses (Figures 5h and S40, Tables S29 and S30). The XPS (Figure S40) resembled that of the individual reaction described above.

The second-generation CE reactions were not limited to the CdS/Cu_2-x_S films as we also performed CE with CdS/Ag_2_S films using Mn^2+^ as the guest cation. Here, the host film was introduced to a solution of 0.2 mmol of MnI_2_ dissolved in 10 mL of acetonitrile and allowed to react at room temperature for 15 min under nitrogen flow. SEM imaging showed that the morphology remained unchanged pre- and postexchange (Figure S41a). EDS and ICP-MS analyses in Figure S41b and Table S31 confirmed the presence of Mn which was corroborated with a blue shift apparent in the UV–vis spectrum (Figure S41c). The faster reaction time in comparison to the exchange with the CdS/Cu_2-x_S template can be rationalized as Ag_2_S has a lower lattice energy in comparison to Cu_2_S meaning it is less stable and thus allowing CE to occur more readily.^11^ Analogous to the CE reactions with Cu_*x*_Se_*y*_, the above-mentioned second-generation reactions were able to proceed with just the halide guest precursor dissolved in acetonitrile, without the addition of a trialkylphosphine. These results further corroborate our reaction modality and the flexibility allotted with the thin film model system.

## Conclusion

In summary, CE reactions can be readily translated into metal-chalcogenide thin films under mild conditions. This was demonstrated using CdS and Cu_*x*_Se_*y*_ model systems fabricated with CBD and thermal coevaporation, respectively. Exchanges were successfully performed by introducing host material to a single cation or multiple cations of Cu^+^, Ag^+^, Cu^2+^, Cd^2+^, Zn^2+^, and Mn^2+^, in either a simultaneous or sequential fashion, to tune the compositional complexity of the material. CE proceeded in both sulfide and selenide systems without notable differences despite the differing fabrication techniques and different morphologies. The CE reactions observed from Cu_*x*_Se_*y*_ and CdS/Cu_2-x_S with harder cations proceeded under simplified reaction conditions without the addition of a trialkyphosphine and at relatively lower reaction temperatures than those employed in colloidal CE reactions. Moreover, it was demonstrated that the exchange between CdS and Ag could be executed using precursor salts with varying counterions and solvents ranging in polarity. These results validate the flexibility and versatility provided using ligand-free thin films. Applications of this work could aid in the creation of design principles through fundamental comparison of exchange reactions under a wide range of conditions, aided by machine learning methods. Thin films are amenable to and compatible with device fabrication frameworks, facilitating translation of this fundamental research into applications in photovoltaics, (photo)electrocatalysis, and optoelectronics.

## Experimental Details

### Reagents

Cadmium sulfate hydrate (≥99.995%), thiourea (≥99.0%), tetrakis(acetonitrile)copper(I) hexafluorophosphate (Cu(CH_3_CN)_4_PF_6_, 97%), copper(II) acetate (98%), silver trifluoromethanesulfonate (triflate, ≥99%), silver tetrafluoroborate (TFB, 98%), and silver *p*- toluenesulfonate (p-TS, ≥ 99%) were purchased from Sigma-Aldrich. Cadmium sulfate hydrate (Puratronic, 99.996%), ammonium hydroxide (28–30 wt %), acetonitrile (ACN, 99.9% Extra Dry), methanol (99.9% Extra Dry), ACN (Optima), 2-propanol (IPA), acetone, ethanol (EtOH, 200 proof, anhydrous), methanol (MeOH, Optima), toluene (certified ACS), tetrahydrofuran (THF, stabilized, 99.9% Extra Dry), zinc bromide (anhydrous, 98%), and nitric acid (65–70%, ICP-OES for trace metal analysis) were purchased from Fisher Scientific. Silver nitrate (99.9%-Ag), silver neodecanoate (min 97%), silver trifluoroacetate (TFA, min 98%), cadmium iodide (99%), and manganese iodide (anhydrous 98+%) were purchased from Strem. Ultrapure water was dispensed from a Millipore Sigma Biopak Polisher system. All reagents were used as received without further purification.

### Thin Film Fabrication

Cadmium sulfide thin films were fabricated via chemical bath deposition (CBD) based on modified literature procedures.^43,44^ Glass microscope slides were cleaned by sequentially sonicating in acetone, IPA, and DI water for five min each and dried with N_2_ gas. One side was then covered with Kapton tape to limit growth to only one side. A 100 mL beaker containing 65.4 mL of ultrapure water was placed in a water bath set to 67 °C and stirred at 700 rpm. Once the water bath temperature equilibrated, 8.9 mL of a 0.015 M cadmium sulfate hydrate aqueous solution and 6.6 mL of 28–30% ammonium hydroxide were added to the beaker. The substrate was placed diagonally into the solution, and after 1.5 min, 4.4 mL of a 0.75 M thiourea aqueous solution was added. The reaction was allowed to proceed for a total of 20 min in a fume hood. Upon completion, the films were rinsed, sonicated in DI water to remove any loose particles, and dried with N_2_. Copper selenide thin films were fabricated on glass microscope slides and FTO-coated glass substrates by employing thermal evaporation. Thermal evaporation was carried out using individual boron nitride bottles with Cu and Se temperatures set to 1360 and 315 °C, respectively, while the substrate temperature was kept at 580 °C with an open shutter for 2.5 min. It is worth mentioning that most CE reactions herein employed thin films on glass substrates. FTO was used as a proof of concept that this technique can also proceed on a conductive substrate for easier translation to optoelectronic and photoelectrocatalytic applications. Results for CE reactions performed on films deposited on FTO can be found in Figures S1, S4 and S5.

### First-Generation CE from CdS

All reactions were carried out with 2.4 cm × 2.5 cm CdS thin films on glass substrates. The exchange of CdS with Ag^+^ was based on an existing literature procedure.^12,52^ In a typical exchange, 0.2 mmol of silver nitrate was dissolved in 20 mL of acetonitrile in a glass jar with a stir bar. Once dissolved, the CdS film was lowered into the solution, and the reaction was allowed to proceed for 1 h under ambient conditions while stirring at 700 rpm. Similarly, the exchange with Cu^2+^ was executed by lowering the CdS film into a solution of 0.2 mmol copper(II) acetate dissolved in 30 mL of water in a glass jar equipped with a stir bar and allowed to proceed for 3 h. The exchange of CdS with Cu^+^ was adapted from a modified literature procedure and upscaled.^54^ In a typical reaction, 1.25 mmol of Cu(CH_3_CN)_4_PF_6_ was dissolved in 250 mL of methanol in a 600 mL beaker equipped with a stir bar in a nitrogen-filled glovebox. Eight CdS thin film samples in a custom-built substrate holder were lowered into the solution. The flask was covered with a lid, and the exchange was allowed to proceed for 1 h at room temperature while stirring at 400 rpm. These films were further used as templates for second-generation exchanges.

### Second-Generation CE from CdS

All second-generation CE reactions were performed by utilizing the CdS first-generation templates described above. The CdS/Cu_2-x_S exchange with Ag^+^ was carried out by submerging the host film in a glass jar containing 10 mL of acetonitrile and 0.2 mmol AgNO_3_ for 1 min under ambient conditions based on a modified literature procedure.^42^ CdS/Cu_2-x_S exchanges with Mn^2+^ and Zn^2+^ were all executed identically. The host film was placed parallel to the bottom of a 50 mL pear-shaped flask with a 29/48 neck in a solution of their respective (0.2 mmol) guest precursor salt (CdI_2_, MnI_2_, or ZnBr_2_) in 10 mL of extra-dry acetonitrile and equipped with a triangle-shaped stir bar. All solutions were prepared and capped with a septum in a nitrogen filled glovebox to avoid oxygen exposure. The flask was connected to a Schlenk line in flow configuration by a needle and heated with a Glascol heating mantel connected to a temperature controller with a thermocouple inserted in the flask. Each reaction was allowed to proceed for 1 h at 70 °C. Second-generation simultaneous exchanges of CdS/Cu_2-x_S with binary guest cations (Mn/Cd and Mn/Zn) were carried out following the same procedure as their individual reactions except 0.1 mmol of each cation was used. Likewise, an individual exchange reaction between CdS/Ag_2_S and Mn^2+^ was executed using the same reaction setup as its CdS/Cu_2-x_S counterpart but at room temperature for a 15 min duration.

### First-Generation CE with Cu_*x*_Se_*y*_

Individual exchange reactions of Cu_*x*_Se_*y*_ with Zn^2+^ and Cd^2+^ were performed following the same procedure as that described above for the CdS/Cu_2-x_S exchanges with Cd^2+^, Mn^2+^, and Zn^2+^. Here, the reaction was carried out with 0.2 mmol of ZnBr_2_/CdI_2_ dissolved in 10 mL of extra-dry acetonitrile and allowed to react for 2 h at 70 °C under N_2_ flow and stirred at 700 rpm. A two-step sequential reaction with Zn^2+^ and Cd^2+^ was carried out in one pot using the same setup as above to yield Cu_x_Se_y_–Zn-Cd. First, a 0.1 mmol solution of ZnBr_2_ in 10 mL of acetonitrile was allowed to react for 1 h at 70 °C. Afterward, 0.1 mmol of CdI_2_ in 5 mL of acetonitrile was injected into the flask, and the reaction was allowed to proceed for another hour. Solutions were prepared in a N_2_-filled glovebox.

### CdS-Ag Precursor/Solvent Study

To test the flexibility of the thin film CE, the reaction with Ag^+^ with CdS was carried out using various salts and solvents. In all reactions, 0.5 mmol of the silver salt was dissolved in 20 mL of solvent in 40 mL of septum-capped vials equipped with a stir bar. A 1.5 cm × 2.5 cm CdS film was lowered into the solution with an alligator clamp with a titanium wire and held in solution by the septum. All solutions were stirred at 300 rpm and allowed to react for 1 h at room temperature. In the precursor study, silver salts of tetrafluoroborate (TFB), *p*-toluenesulfonate (p-TS), trifluoracetate (TFA) (neodec), triflate, and nitrate were dissolved in methanol, while neodecanoate, TFB, triflate, and TFA salts were dissolved in toluene. In the solvent study, silver trifluoroacetate was dissolved in toluene, THF, acetonitrile, ethanol, methanol, and Millipore water.

### Characterization Methods

All films were thoroughly rinsed with the solvent utilized in their respective reactions to make sure any excess precursor salt was removed before proceeding to characterization. SEM was utilized to monitor the thin film morphology before and after the CE reaction and for elemental analysis in conjunction with EDS using SEM/FIB Auriga 60. All images were taken at an accelerating voltage of 3.00 kV with a secondary electron secondary ion detector, a focused ion beam probe of 30 kV:50 pA, and a scan speed of 9. EDS spectra were taken under the same conditions with an accelerating voltage of 12.00 kV. Prior to analysis, samples were cut to a dimension of 1 cm × 1 cm and sputtered with Au/Pd for 35 s to avoid charging utilizing a Denton Vacuum, Desk IV sputter. It is worth noting that there are elements present in the EDS spectra that are not related to the sample, such as Au and Pd, due to sputtering. In addition, Si, Ca, O, Na, Mg, K, Sn, Si, and O are present from the microscope slide and FTO substrate, respectively. Absorbance spectra of the thin films were acquired pre- and postexchange using a Jasco V-770 spectrophotometer equipped with an integrating sphere. Spectra were taken from a range of 350–1200 nm at a scan speed of 400 nm/min with a data interval of 0.5 nm. ICP-MS was used for quantification of cations of host material and CE products using Agilent Technologies 7500 Series ICP-MS and Thermo iCAP ICP-TQ-MS. Samples were prepared by digesting a 1 cm × 1 cm piece of film in 2 mL of trace metal grade nitric acid for all CdS samples and 5 mL for Cu_*x*_Se_*y*_ samples. All samples were then diluted with ultrapure water to a 2.8% (v/v) nitric acid matrix prior to measurement. Ion concentration was determined through a calibration curve ranging from 0 to 1000 ppb. It should be noted that we were not able to monitor sulfur in ICP-MS analysis due to the low sensitivity, as the first ionization potential is high. GIXRD was utilized to monitor the crystallinity and identify product phases using Rigaku Thin Film HRXRD with Cu Kα_1_ X-ray source (λ = 1.54 Å), an operating voltage of 40 kV, and a current of 50 mA with an incidence angle of 0.5°. AFM and Raman spectroscopy were employed to investigate the thin film uniformity, surface roughness, and Raman activity pre- and postreaction using AFM_SmartSPM 1000 + Raman_LabRAM HR Evolution. AFM analysis was run on noncontact mode, while Raman was conducted with a 532 nm laser with a grating of 1800. XPS was employed for surface quantification and to track oxidation states using XPS Thermo Scientific K-alpha with a monochromatic Al Kα X-ray source with a spot size of 400 μm, 10 scan accumulation, a pass energy of 50 eV, a dwell time of 50 ms, and an energy step of 0.1 eV.

### General Safety Considerations

The CBD reaction of CdS produces ammonia fumes that can be harmful. As such, the reaction should always be conducted in a fume hood to prevent exposure. Cadmium is a toxic heavy metal; appropriate PPE should be worn when handling this and all other reagents/solvents. THF (used in the precursor/solvent study) is a known peroxide forming solvent; it should be stored in a cool, dark place and capped tightly. Before using, we should inspect the shelf life and the bottle for any crystal formation (could indicate peroxidation). We should handle it under an inert atmosphere. Nitric acid (used in sample digestion for ICP) is highly corrosive.

## Supporting Information Available

SEM images, EDS, UV–vis, ICP-MS, Raman, AFM imaging, AFM *R*_ms_ statistics, XPS surface scans, and XPS quantification of pristine CdS and pristine Cu_*x*_Se_*y*_ films; raw ICP-MS data, Raman spectra, AFM imaging, AFM *R*_ms_ statistics, XPS surface scans, and XPS quantification of CdS, CdS/Cu_2-x_S, and Cu_*x*_Se_*y*_ CE reactions, and UV–vis of Cu_*x*_Se_*y*_ and CdS/Cu_2-x_S CE reactions; characterization for the CE reaction between CdS and Cu^2+^, Cu_*x*_Se_*y*_ attempted the one-pot CE reaction with Zn and Cd and Cu_*x*_Se_*y*_ exchange with Ag, and CdS/Ag_2_S exchange with Mn (PDF)
