# Correction to “Flexible Cation Exchange Environment via Ligand-Free Metal Chalcogenide Thin Films”

**DOI:** 10.1021/acsnanoscienceau.5c00021

**Published:** 2025-03-27

**Authors:** Hannah R. Lacey, Kevin D. Dobson, Emil A. Hernández-Pagán

The authors would like to correct an unintentional omission in the acknowledgments for which we apologize. We thank John Routzahn and Prarthona Zaman for their help in early stages of the reactions and experimental design, plus proofreading the first manuscript draft. This correction does not affect the data and conclusions presented in the published article.

