# Supplementary material for: Flexible Cation Exchange Environment via Ligand-Free Metal Chalcogenide Thin Films
Source: ACS Nanosci Au. 2024 Nov 8;5(1):9–20. doi: 10.1021/acsnanoscienceau.4c00023 (PMC11843514; doi:10.1021/acsnanoscienceau.4c00023)
Supplement: Supplementary file 1 — ng4c00023_si_001.pdf [file ng4c00023_si_001.pdf]

Supporting Information

Flexible cation exchange environment via ligand-free metal chalcogenide thin films

Hannah R. Lacey[1], Kevin D. Dobson[2], Emil A. Hernández- Pagán[1]*

*Corresponding author: emilhp@udel.edu

[1]Department of Chemistry and Biochemistry, University of Delaware, Newark, Delaware 19716, United States

[2]Institute of Energy and Conversion, University of Delaware, Newark, Delaware 19716, United States

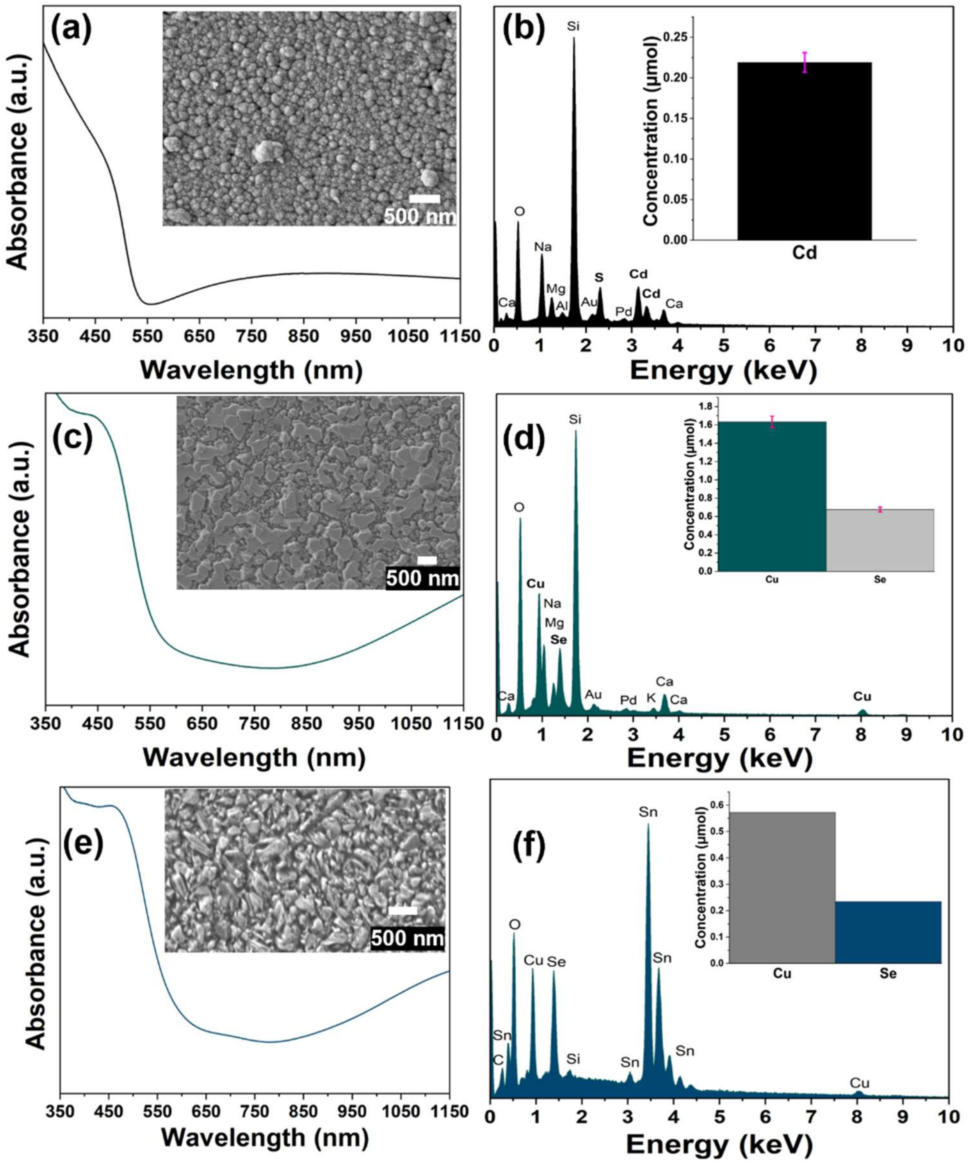

Figure S1: (Left column) UV-Vis spectra of (a) pristine CdS grown on a microscope slide, (c) pristine Cu_xSe_y grown on microscope slide and e) pristine Cu_xSe_y grown on FTO with insets of SEM images showing their respective morphologies. (Right column) SEM-EDS spectra showing the elemental composition of (b) CdS, (d) Cu_xSe_y and Cu_xSe_y on FTO with insets corresponding to the concentrations obtained from ICP-MS with error bars representing the standard error of the mean. Au and Pd present in the EDS spectra were due to sputtering used to avoid charging during imaging and the other elements (i.e. Si, Ca, O, Na, Mg, K and Sn, Si, O) are present from the microscope slide and FTO substrate respectively. These elements were observed in all of the EDS spectra provided herein.

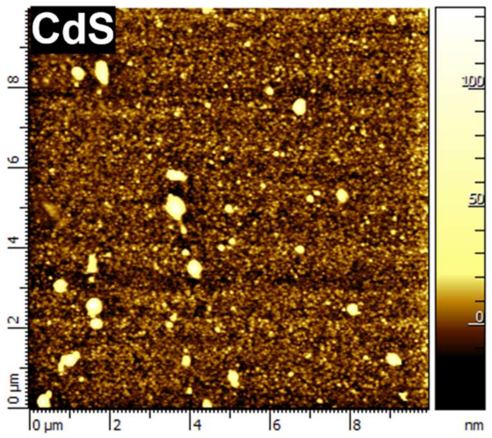

Figure S2: Representative two-dimensional AFM image of pristine CdS films grown by chemical bath deposition.

Sample	Region 1	Region 2	Region 3	Region 4	Region 5	Average	St. Dev	St. Error
**CdS**	13.3	10.7	13.6	12.2	11.1	**12.2**	1.1	0.5
**$CdS/Cu_{2-x}S$**	9.8	6.9	6.9	7.0	21.1	**10.3**	5.2	2.1
**CdS/Ag_2S**	19.0	10.6	13.7	15.9	12.5	**14.4**	2.9	1.3
**$CdS/Cu_{2-x}S$-Zn**	9.6	9.0	10.5	14.3	14.2	**11.5**	2.3	1.0
**$CdS/Cu_{2-x}S$-Ag**	10.9	12.1	12.4			**11.8**	0.6	0.4

Table S1: Roughness Mean Square value (R_{ms}) values in nm obtained via AFM analysis of CdS and corresponding CE reactions in accordance with Figures S2 (CdS), S10 (CdS/Ag_2S), S14 ($CdS/Cu_{2-x}S$), S32 ($CdS/Cu_{2-x}S$-Ag), and S34 ($CdS/Cu_{2-x}S$-Zn). Statistics were calculated on 5 regions of an area of 10 mm x 10 mm and averaged for each sample apart from $CdS/Cu_{2-x}S$-Ag where 3 regions were analyzed.

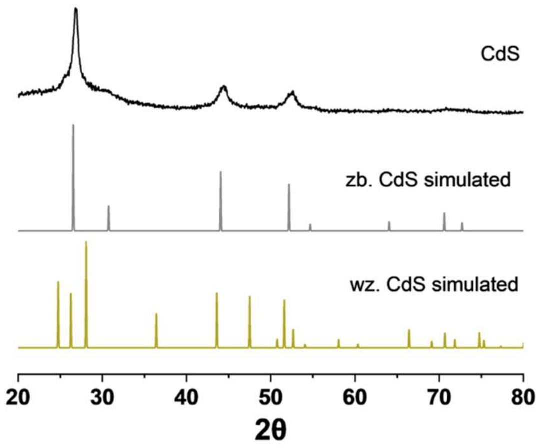

Figure S3: Grazing incidence XRD pattern of pristine CdS film with reflections matching the zincblende CdS reference pattern. The asymmetry of the peak at 26.8° 2θ is due to overlapping reflections from wurtzite CdS. An average crystallite size of 5.7 nm was determined via Scherer analysis by taking the FWHM of the reflection at 44.3° 2θ.

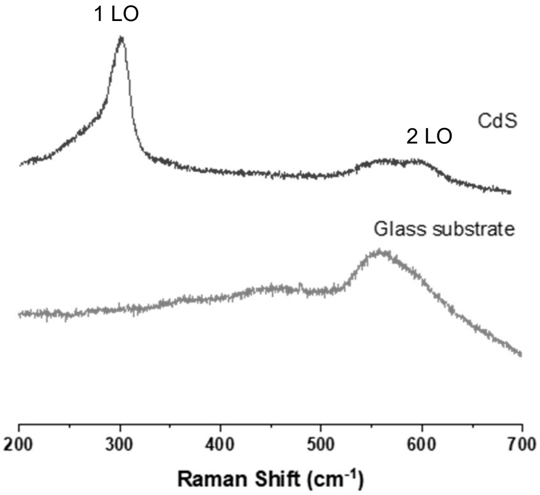

Figure S4: Comparison of the Raman spectra of a bare glass substrate (bottom/gray) and the CdS films grown on glass by chemical bath deposition on glass (top/black). The CdS shows the 1LO and 2LO modes in agreement with literature reports. [1,2]

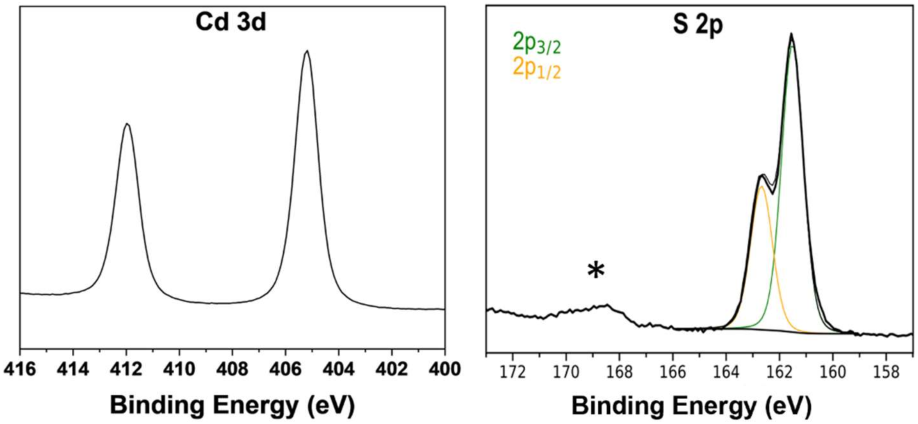

Figure S5: XPS surface scans of pristine CdS. The deconvoluted S 2p spectrum is consistent with that reported for CdS. *Binding energy at 169 eV corresponding to sulfate species from surface oxidation. [3,4]

Sample	Peak	Average Atomic Percentage	St. Dev
CdS	Cd 3d	49.6	0.6
	S 2p	50.4	0.6

Table S2: XPS surface quantification of pristine CdS with the average atomic percentage calculated across three regions of the sample. The average Cd:S ratio is 1.0

Sample	[Cd] µmol
CdS_1	0.214
CdS_2	0.225
CdS_3	0.191
CdS_4	0.255
CdS_5	0.209
CdS_6	0.221
CdS_7	0.200
CdS_8	0.286
CdS_9	0.177
CdS_10	0.205
CdS_11	0.148
CdS_12	0.292
Average	0.219
Standard Deviation	0.040
Standard Error	0.012

Table S3: ICP-MS analysis of CdS host film. Raw data was obtained in units of ppb and converted to µmol for analysis. It should be noted that we were unable to monitor S due to the low sensitivity as the first ionization potential is high. Data from this table was used to make bar graph inset shown in Figure S1b.

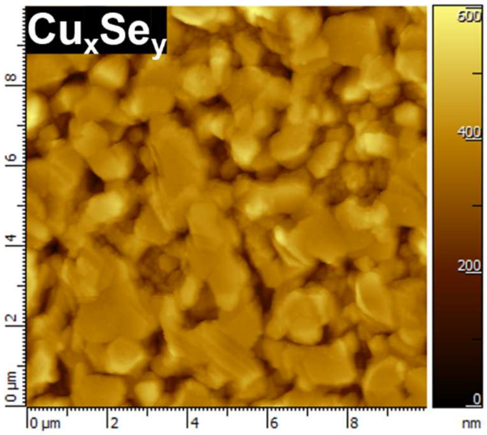

Figure S6: Representative two-dimensional AFM image of pristine Cu_xSe_y film.

Sample	Region 1	Region 2	Region 3	Region 4	Average	St.Dev	St. Error
**Cu_xSe_y**	68.8	68.7	69.0	67.4	**68.5**	0.6	0.3
**Cu_xSe_y-Cd**	61.7	60.7	60.7		**61.1**	0.4	0.3
**Cu_xSe_y-Zn**	65.5	73.0	72.1	59.0	**67.4**	5.6	2.8

Table S4: R_{ms} values in nm obtained via AFM analysis in conjunction with Figure S6 of Cu_xSe_y and CE reactions with Cd (Cu_xSe_y-Cd, Figure S22) and Zn (Cu_xSe_y-Zn, Figure S27). Each sample was measured in 3 or 4, 10 mm x 10 mm regions of the film and averaged.

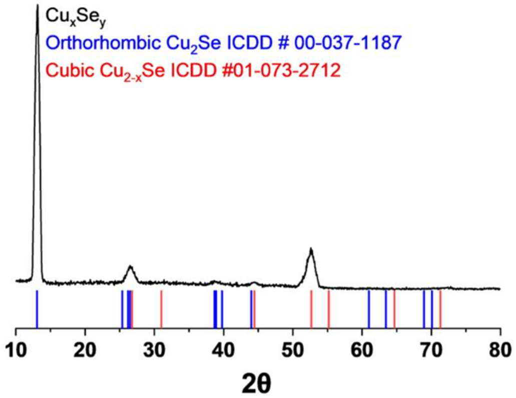

Figure S7: Grazing incidence XRD pattern of pristine Cu_xSe_y film with reflections matching the orthorhombic and cubic copper selenide reference patterns. An average crystallite size of 10.2 nm determined via Scherer analysis by taking the FWHM of the orthorhombic peak at 13.0° 2θ and 6.0 nm based on the peak at 52.6°2θ corresponding to the cubic structure.

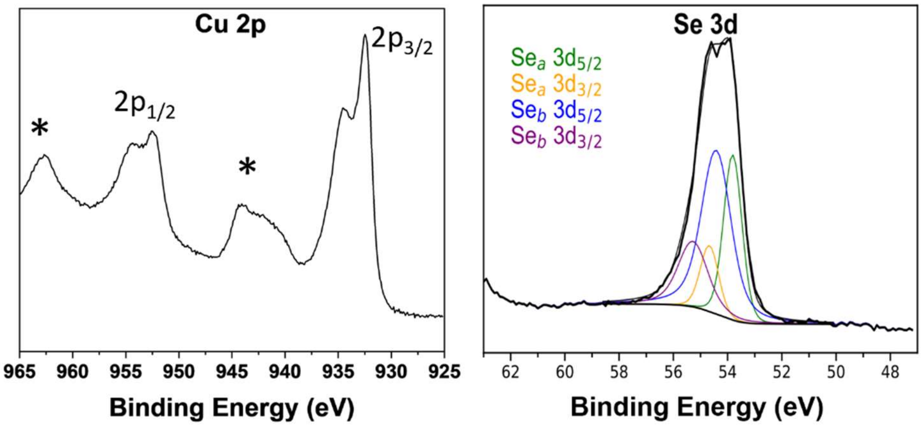

Figure S8: XPS surface scans of pristine Cu_xSe_y. *Satellite feature corresponding to Cu^{2+}.[3,5] The deconvoluted Se 3d spectrum shows the presence of a selenide with likely other selenium species.[3]

Sample	Cu µmol	Se µmol	Cu:Se
Cu_xSe_y	1.719	0.714	2.407
Cu_xSe_y	1.545	0.637	2.425
**Average**	**1.632**	**0.676**	**2.416**
St. Dev	0.087	0.039	0.009
St. Error	0.061	0.027	0.006

Table S5: ICP-MS analysis of the pristine Cu_xSe_y films digested using nitric acid. Raw data was obtained in units of ppb and converted to µmol for analysis. ICP-MS analysis of the first-generation exchange between Cu_xSe_y and various cations used to create bar graph insets in Figure S1d.

Sample	Peak	Average Atomic Percentage	St. Dev
Cu_xSe_y	Cu 2p3	64.4	1.7
	Se 3d	35.6	1.7

Table S6: XPS surface quantification pristine Cu_xSe_y with the average atomic percentage calculated across three regions of the sample. The average Cu:Se is 1.8.

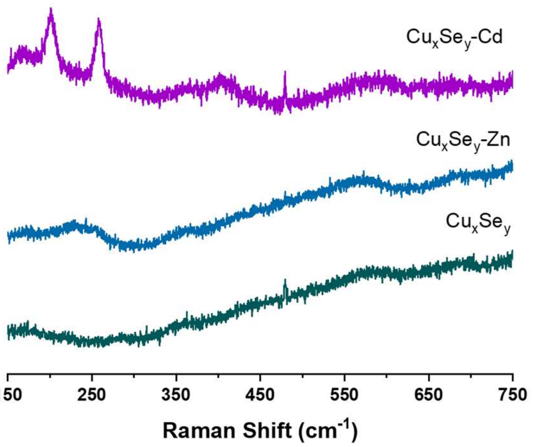

Figure S9: Raman Spectra obtained for pristine Cu_xSe_y films (bottom) and after exchange reactions with Zn^{2+} (Cu_xSe_y-Zn, middle) and Cd^{2+} (Cu_xSe_y-Cd, top). The Cu_xSe_y-Cd spectra show peaks at 203 cm^{-1}, 260 cm^{-1}, and 404 cm^{-1} which have been reported as the 1LO mode of zincblende CdSe, Se-Se bonds vibration, and 2LO of CdSe, respectively.[6,7]

Time (mins)	Average [Cd] µmol	Average [Ag] µmol	Ag:Cd
0	0.216	0.001	0.002
5	0.178	0.095	0.532
30	0.138	0.197	1.432
60	0.102	0.221	2.164
90	0.084	0.252	3.001
120	0.085	0.372	4.394

Table S7: ICP-MS analysis on the CE reaction between CdS films and Ag^+ (CdS/Ag_2S), tracking the reaction progress through 120 minutes. Cation concentrations were averaged over 3 samples to obtain the Ag:Cd ratio. The time study was used to determine a generalizable reaction length. For all reactions carried out with the CdS host film, the reaction time was maintained at 1 hour as that is when we determined sufficient exchange to occur as the [Ag] is double that of [Cd]. Raw data was obtained in units of ppb and converted to µmol for analysis.

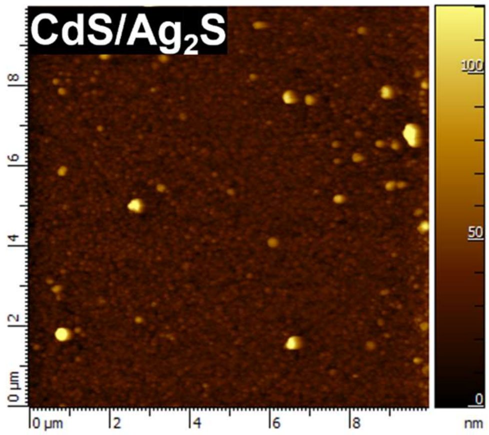

Figure S10: Two-dimensional AFM image obtained after the cation exchange reaction between CdS film and Ag^{+} (CdS/Ag_2S). No noticeable differences were observed on the topography with an R_{ms} of 14.4 ± 2.9 nm, close to the 12.2 ± 1.1 nm for the pristine CdS films. See Table S1 for R_{ms} data.

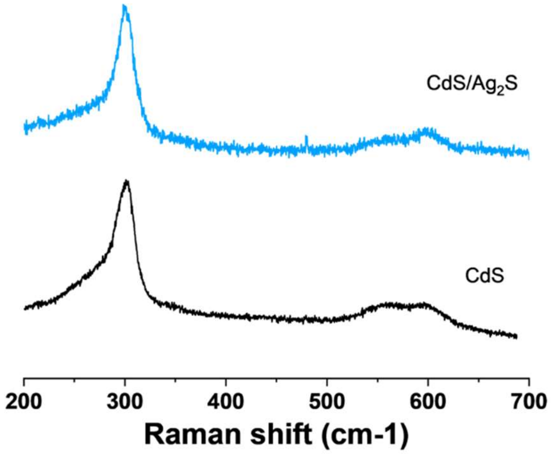

Figure S11: Raman spectra of a pristine CdS film (bottom) and a film after cation exchange reaction between CdS and Ag^{+} (CdS/Ag_2S, top). The fact that the spectrum does not change is indicative of a partial exchange with CdS still present in the sample. This observation is consistent with the grazing incidence XRD shown in Figure S13.

Sample	[Cd] µmol	[Ag] µmol	Ag:Cd
CdS-Ag_1	0.075	0.233	3.113
CdS-Ag_2	0.089	0.204	2.298
CdS-Ag_3	0.072	0.269	3.748
Average	0.078	0.235	
Standard Deviation	0.007	0.027	
Standard Error	0.004	0.015	

Table S8: ICP-MS analysis by digestion in nitric acid of three first-generation samples of the exchange between CdS films and Ag^+ (CdS/Ag_2S). This data was used to create bar graph insets in Figure 1e of the main text. Raw data was obtained in units of ppb and converted to µmol for analysis.

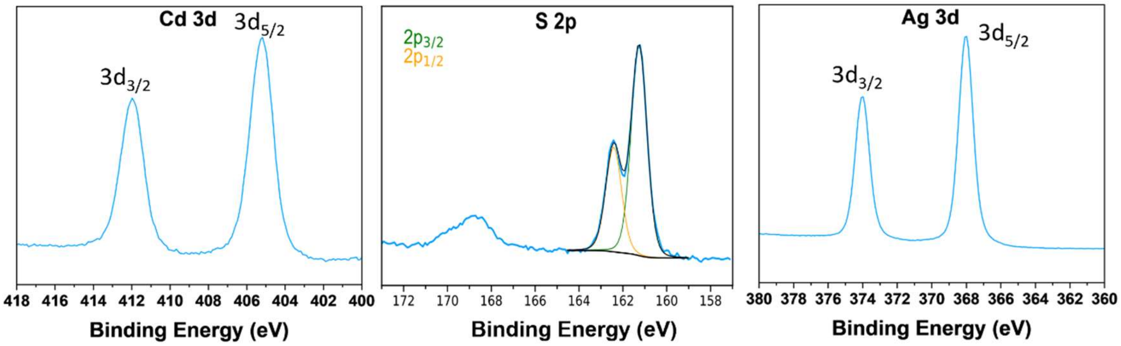

Figure S12: XPS surface scans of CdS/Ag_2S. The deconvoluted S 2p spectrum is consistent with that reported for a metal sulfide. Binding energy at 169 eV corresponding to sulfate species. The Ag $3d_{5/2}$ and $3d_{3/2}$ peaks of 368.6 eV and 374.6 eV are in agreement with Ag^+.[3,8]

Sample	Peak	Average Atomic Percentage	St. Dev
CdS/Ag_2S	Cd 3d	15.6	0.7
	Ag 3d	51.2	0.4
	S 2p	33.2	0.9

Table S9: XPS surface quantification of a first-generation sample where a CdS film was exchanged with Ag^+ (CdS/Ag_2S). The average atomic percentage calculated across three regions of the sample.

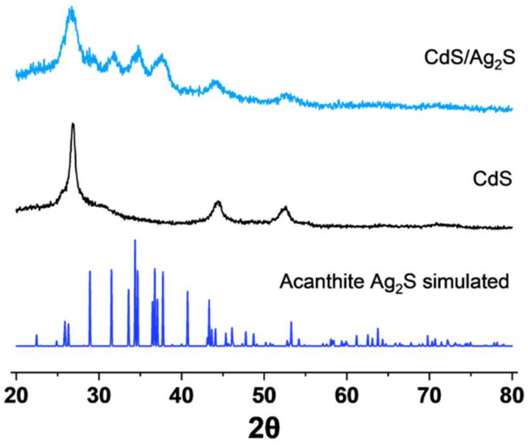

Figure S13: Grazing incidence XRD patterns first-generation sample where a CdS film was exchanged with Ag^+ (CdS/Ag_2S, top). The broadness and low intensity of the reflections makes unambiguous identification difficult, however there is some overlap with the reference for acanthite Ag_2S, which is a known stable polymorph.[9] The pattern for a pristine CdS film (middle) is included for comparison.

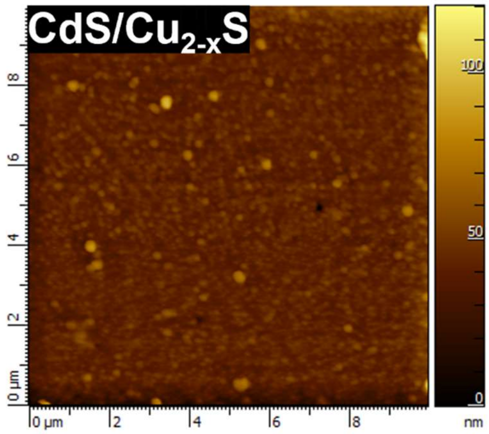

Figure S14: Two-dimensional AFM image obtained after the cation exchange reaction between a CdS film and Cu^+ ($CdS/Cu_{2-x}S$). No noticeable differences were observed on the topography with an R_{ms} of 10.3 ± 5.2 nm, close to the 12.4 ± 1.2 nm for the pristine CdS films. See Table S1 for R_{ms} data.

Sample	[Cd] μmol	[Cu] μmol	Cu:Cd
CdS-Cu(I)_1	0.016	0.342	21.815
CdS-Cu(I)_2	0.015	0.310	21.179
CdS-Cu(I)_3	0.062	0.786	12.699
CdS-Cu(I)_4	0.244	0.470	1.925
CdS-Cu(I)_5	0.039	0.361	9.305
CdS-Cu(I)_6	0.031	0.327	10.557
CdS-Cu(I)_7	0.034	0.315	9.360
CdS-Cu(I)_8	0.071	0.435	6.162
Average	0.064	0.418	
Standard Deviation	0.071	0.149	
Standard Error	0.025	0.053	

Table S10: ICP-MS analysis by digestion in nitric acid of three first-generation samples where CdS films were exchanged with Cu^+ ($CdS/Cu_{2-x}S$). This data was used to create bar graph insets in Figure 1f of the main text. Raw data was obtained in units of ppb and converted to μmol for analysis.

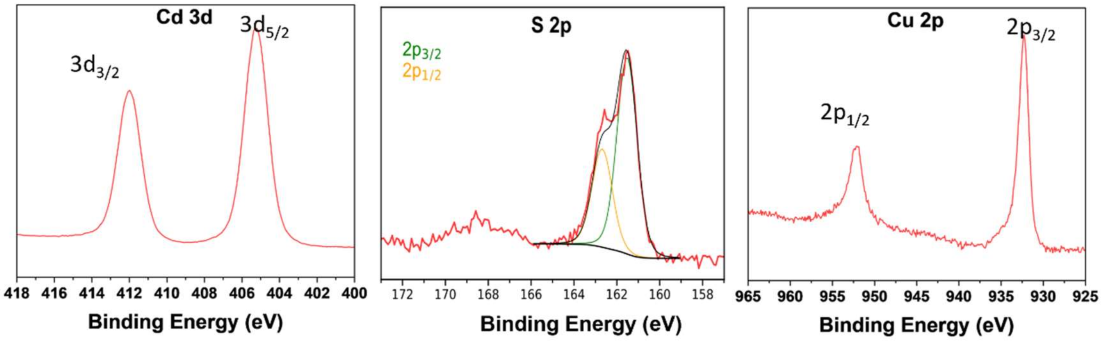

Figure S15: XPS surface analysis of a first-generation sample of the exchange between CdS films and Cu^+ ($CdS/Cu_{2-x}S$). The deconvoluted S 2p spectrum is consistent with that reported for a metal sulfide. Binding energy at 169 eV corresponding to sulfate species. Cu $2p_{3/2}$ and $2p_{1/2}$ peaks of 932.3 eV and 952.2 eV, and no satellite peaks, as expected for Cu^+.[3–5]

Sample	Peak	Average Atomic Percentage	St. Dev
$CdS/Cu_{2-x}S$	Cd 3d	62.6	7.0
	Cu 2p3	16.4	5.9
	S 2p	21.0	6.8

Table S11: XPS surface quantification of a first-generation sample of the exchange between CdS films and Cu^+ ($CdS/Cu_{2-x}S$) with the average atomic percentage calculated across three regions of the sample. The data indicates an accumulation of Cd^{2+} at the surface.

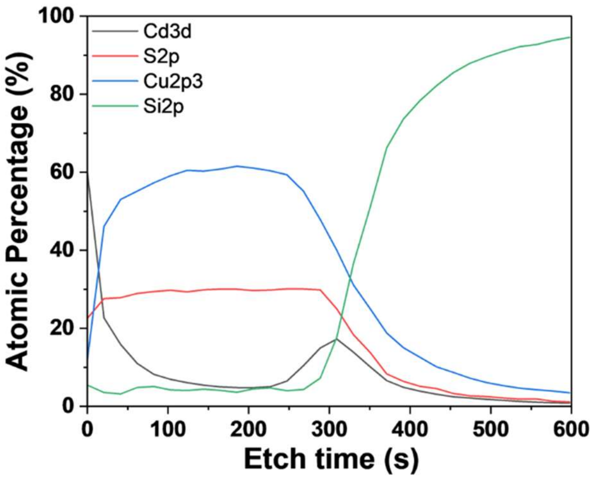

Figure S16: XPS depth profile of a first-generation sample of the exchange between CdS films and Cu^{+} ($CdS/Cu_{2-x}S$). The profile shows a Cd^{2+} surface layer is removed within 20 seconds with the Cu^{+} signal increasing thereafter.

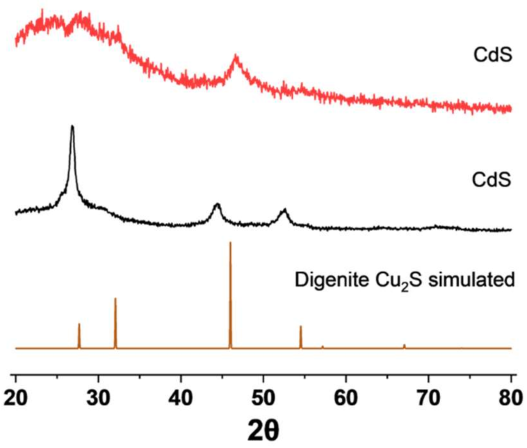

Figure S17: Grazing incidence XRD patterns of a first-generation sample of the exchange between CdS films and Cu^{+} ($CdS/Cu_{2-x}S$, top) and the pristine CdS film (middle). The $CdS/Cu_{2-x}S$ pattern shows the disappearance of the CdS reflections and appearance of one broad reflection at 46.7° which potentially matches the 220 plane of the reference pattern for digenite copper sulfide.

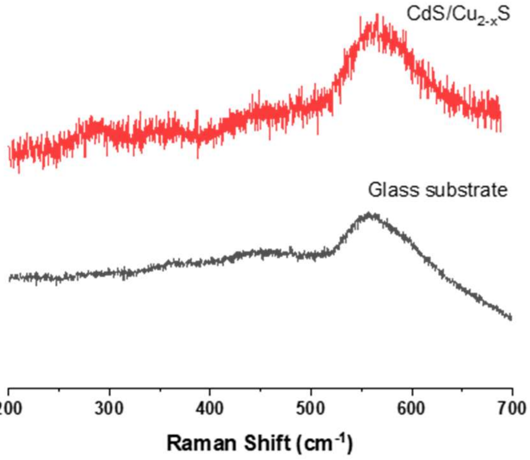

Figure S18: Raman spectra of $CdS/Cu_{2-x}S$ (top) in comparison with bare glass substrate (bottom) showing there the film is no longer Raman active post cation exchange with Cu^+.

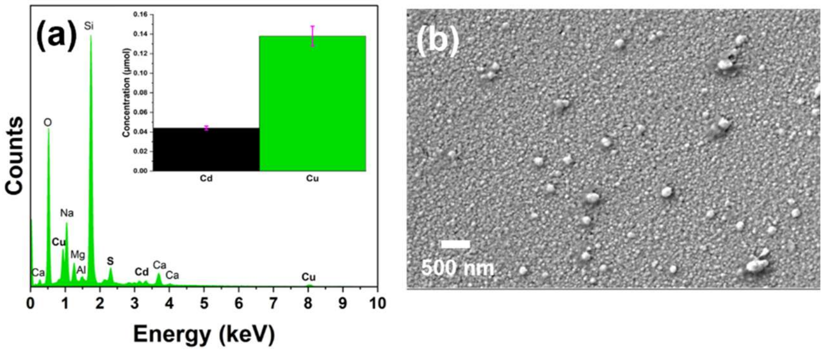

Figure S19: Exchange reaction between CdS films and Cu2+ in water. (a) SEM-EDS spectrum with bar graph insets corresponding to the cation concentrations obtained from ICP-MS analysis of digested films after exchange and (b) SEM image showing the morphology after the exchange. Note: The error bars on the inset are representative of the standard error of the mean obtained from the averaging of three trials (see Table S12).

Sample	[Cd] µmol	[Cu] µmol	Cu:Cd
CdS-Cu(II)_1	0.048	0.161	3.357
CdS-Cu(II)_2	0.044	0.121	2.774
CdS-Cu(II)_3	0.040	0.132	3.324
Average	0.044	0.138	
Standard Deviation	0.003	0.017	
Standard Error	0.002	0.010	

Table S12: ICP-MS analysis by digestion in nitric acid of three first-generation samples of the exchange between CdS films and Cu^{2+} (CdS-Cu(II)) used to create bar graph shown in Figure S19.

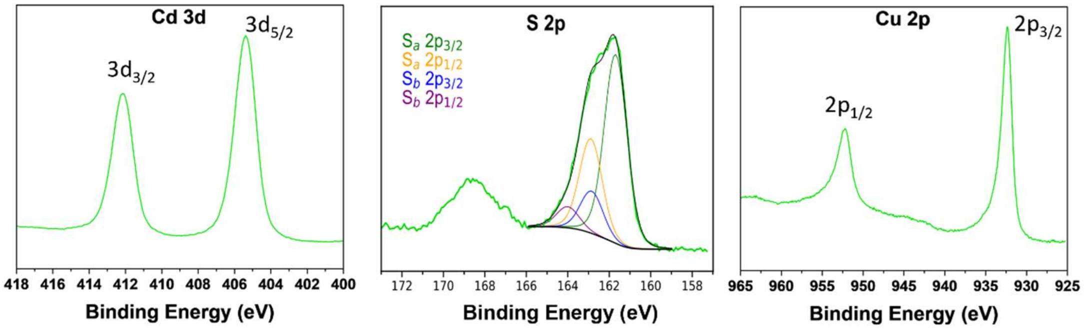

Figure S20: XPS surface scans of a first-generation sample of the exchange between CdS films and Cu^{2+} (CdS-Cu_xS). The deconvoluted S 2p spectrum differs from the other first-generation samples and shows what appears to be oxidized sulfide species in addition to the expected metal sulfide. The intensity of the binding energy at 169 eV corresponding to sulfate species is also greater than the other first-generation samples. The Cu 2p spectrum aligns with that expected for Cu^{+} and does not show satellite peaks expected for Cu^{2+}.[3,5]

Sample	Peak	Average Atomic Percentage	St. Dev
CdS/Cu_xS	Cd 3d	24.0	0.0
	Cu 2p3	22.0	2.2
	S 2p	54.0	2.2

Table S13: XPS surface quantification of a first-generation sample of the exchange between CdS films and Cu^{2+} (CdS-Cu_xS) with the average atomic percentage calculated across three regions of the sample.

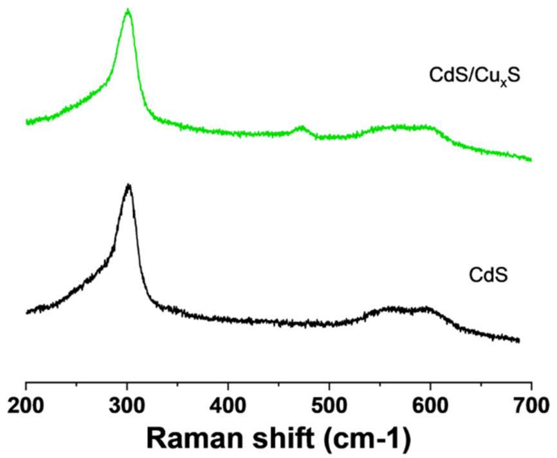

Figure S21: Raman spectrum obtained for a first-generation sample of the exchange between CdS films and Cu^{2+} (CdS-Cu_xS, top) compared to that of a pristine Cd film (bottom). The resemblance between the spectra suggests a lesser degree of exchange with CdS remaining after the exchange. The spectrum for CdS-Cu_xS (top) shows the emergence of a mode at 475 cm^{-1} which literature reports attribute to S-S bonds that result from oxidation. [10,11]

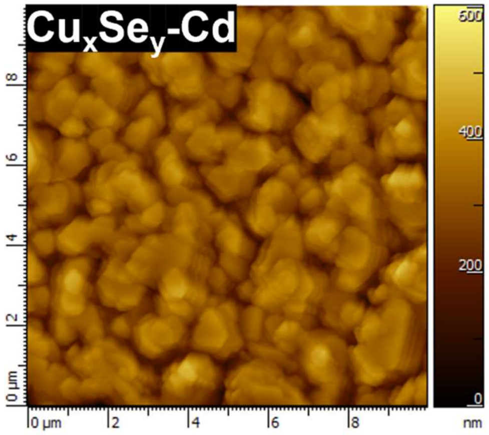

Figure S22: Two-dimensional AFM image obtained after the cation exchange reaction between Cu_xSe_y films and Cd^{2+} (Cu_xSe_y-Cd). No noticeable differences were observed on the topography with an R_{ms} of 61.1 ± 0.1 nm, close to the 68.5 ± 0.6 nm for the pristine Cu_xSe_y films. See Table S4 for R_{ms} data.

Sample	Cu µmol	Cd µmol	Se µmol	Cu:Se	Cd:Cu
Cu_xSe_y-Cd-1	0.793	0.488	0.748	1.060	0.616
Cu_xSe_y-Cd-2	1.026	0.552	0.916	1.120	0.538
**Average**	**0.909**	**0.520**	**0.832**	**1.090**	**0.577**
St. Dev	0.117	0.032	0.084	0.030	0.039
St. Error	0.082	0.023	0.059	0.021	0.027

Table S14: ICP-MS analysis by digestion in nitric acid of two first-generation samples of the exchange between Cu_xSe_y films and Cd^{2+} (Cu_xSe_y-Cd) used to create bar graph shown in Figure 2b.

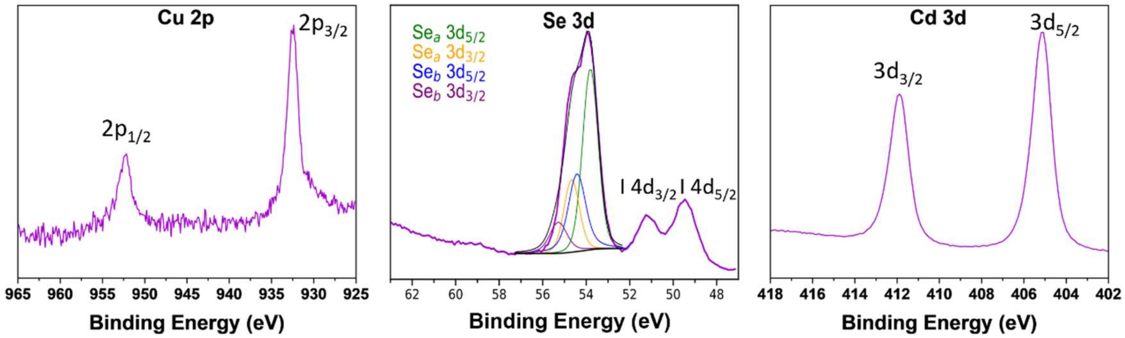

Figure S23: XPS surface analysis of a first-generation sample of the exchange between Cu_xSe_y films and Cd^{2+} (Cu_xSe_y-Cd). The Cu 2p spectrum aligns with that expected for Cu^+ and does not show satellite peaks expected for Cu^{2+}.The deconvoluted Se 3d spectrum shows the presence of a selenide with likely other selenium species. Also seen is a peak corresponding to the iodide from the precursor. The Cd $3d_{5/2}$ and $3d_{3/2}$ binding energies of 405.2 eV and 411.9 eV as expected for Cd^{2+}.[3,4]

Sample	Peak	Average Atomic Percentage	St. Dev
Cu_xSe_y-Cd	Cu 2p3	6.3	1.3
	Se 3d	50.7	0.9
	Cd 3d	43.0	0.7

Table S15: XPS surface quantification of a first-generation sample of the exchange between Cu_xSe_y films and Cd^{2+} (Cu_xSe_y-Cd) with the average atomic percentage calculated across three regions of the sample.

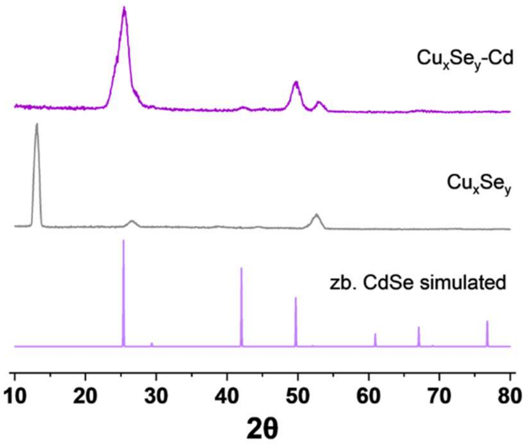

Figure S24: Grazing incidence XRD patterns of a first-generation sample of the exchange between Cu_xSe_y films and Cd^{2+} (Cu_xSe_y-Cd, top) and the pristine Cu_xSe_y film (middle). The Cu_xSe_y-Cd pattern shows the emergence of reflections that match the reference pattern for zincblende CdSe with residual reflections of Cu_xSe_y.

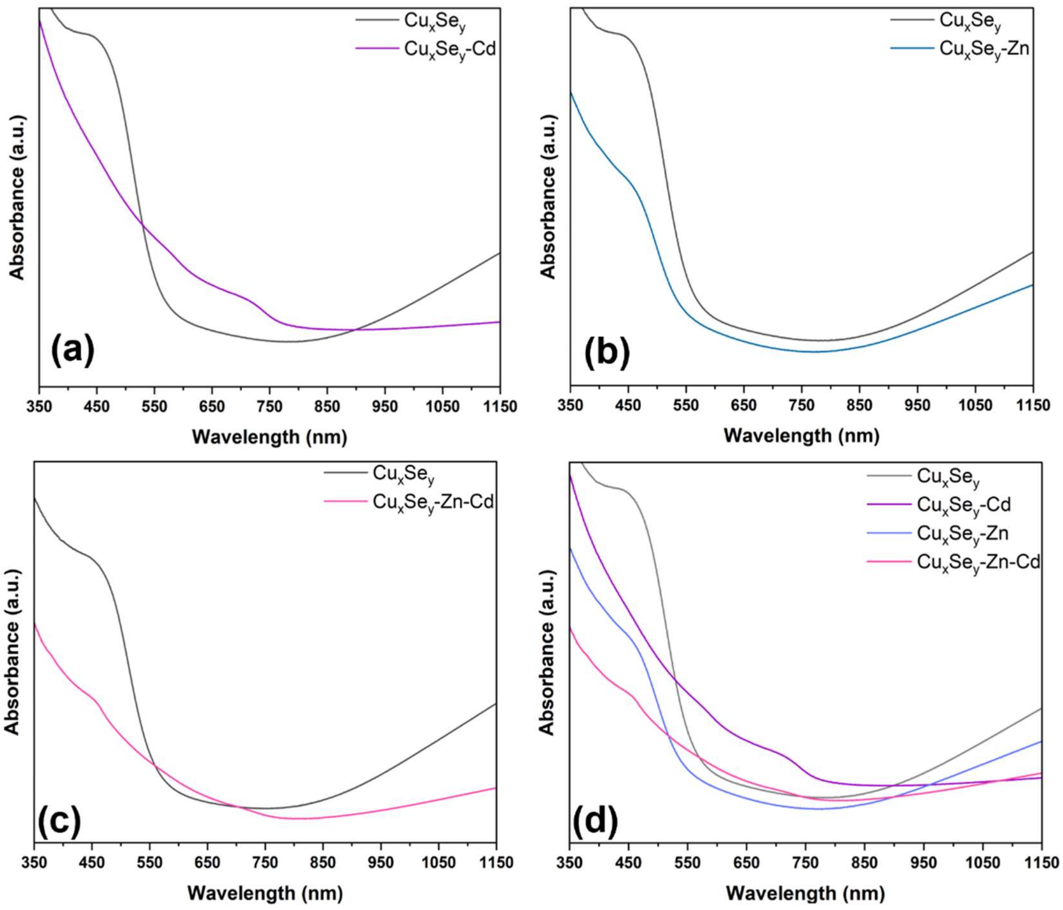

Figure S25: UV-Vis absorbance spectra of first-generation exchanges with respect to Cu_xSe_y host films with (a) Cd^{2+} (b) Zn^{2+} (c) sequential exchange where Zn^{2+} was first introduced to the host film and allowed to react for 1 hour than Cd^{2+} was injected into the pot and allowed to react for another hour. (d) Comparison of individual cation exchange products and sequential exchange products.

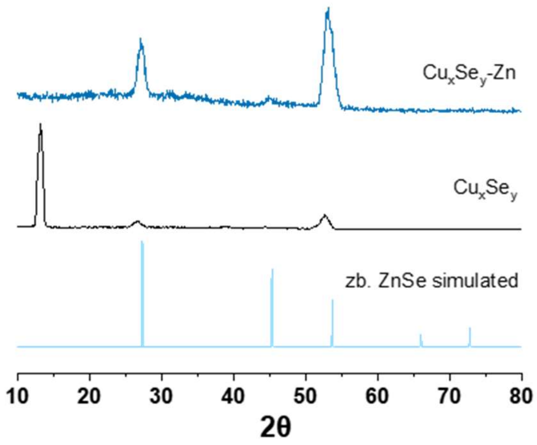

Figure S26: Grazing incidence XRD patterns of a first-generation sample of the exchange between Cu_xSe_y films and Zn^{2+} (Cu_xSe_y-Zn, top) and the pristine Cu_xSe_y film (middle). The Cu_xSe_y-Zn pattern shows the emergence of reflections that match the reference pattern for zincblende ZnSe.

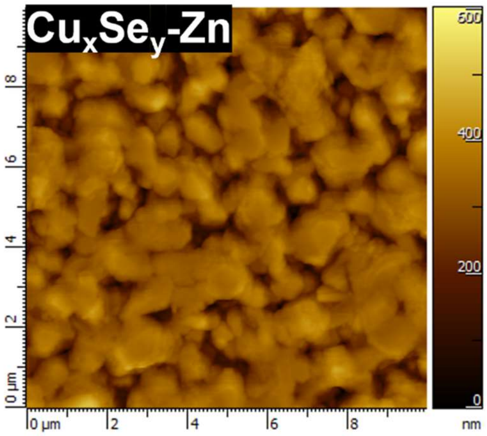

Figure S27: Two-dimensional AFM image obtained after the cation exchange reaction between Cu_xSe_y films and Zn^{2+} (Cu_xSe_y-Zn). No noticeable differences were observed on the topography with an R_{ms} of 67.4 ± 5.6 nm, close to the 68.5 ± 0.6 nm for the pristine Cu_xSe_y films. See Table S4 for R_{ms} data.

Sample	Cu µmol	Zn µmol	Se µmol	Cu:Se	Zn:Cu
Cu_xSe_y-Zn-1	1.315	0.236	0.683	1.926	0.179
Cu_xSe_y-Zn-2	1.693	0.294	0.871	1.945	0.174
**Average**	**1.504**	**0.265**	**0.777**	**1.935**	**0.177**
St. Dev	0.189	0.029	0.094	0.009	0.003
St. Error	0.134	0.021	0.066	0.006	0.002

Table S16: ICP-MS analysis by digestion in nitric acid of two first-generation samples of the exchange between Cu_xSe_y films and Zn^{2+} (Cu_xSe_y-Zn). Used to make bar graph inset in Figure 2d.

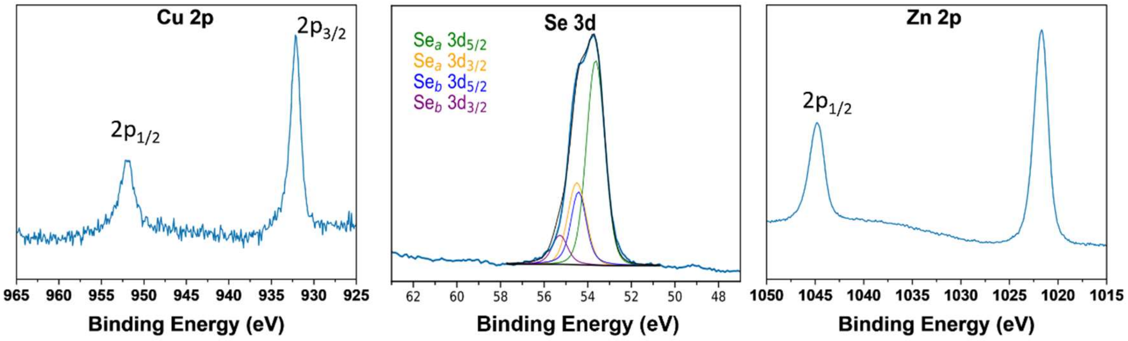

Figure S28: XPS surface analysis of a first-generation sample of the exchange between Cu_xSe_y films and Zn^{2+} (Cu_xSe_y-Zn). The Cu 2p spectrum aligns with that expected for Cu^+ and does not show satellite peaks expected for Cu^{2+}.The deconvoluted Se 3d spectrum shows the presence of a selenide with likely other selenium species. The Zn spectrum is consistent with Zn^{2+} based on a FWHM of value of 1.8 eV for the $2p_{3/2}$ peak.[12] Determination of Zn oxidation state based on the binding energy is difficult as there is great overlap.[3]

Sample	Peak	Average Atomic Percentage	St. Dev
Cu_xSe_y-Zn	Cu 2p3	5.2	1.3
	Se 3d	48.0	0.5
	Zn 2p3	46.8	1.1

Table S17: XPS surface quantification of a first-generation sample of the exchange between Cu_xSe_y films and Zn^{2+} (Cu_xSe_y-Zn) with the average atomic percentage calculated across three regions of the sample.

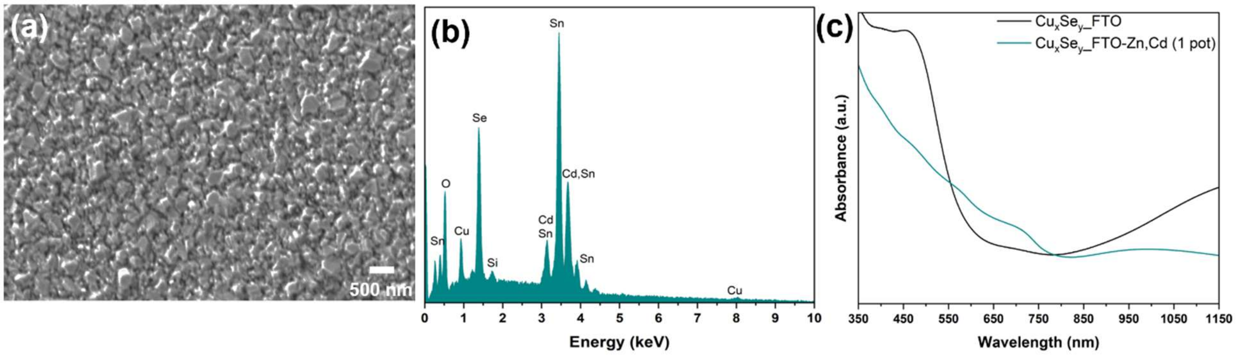

Figure S29: (a) SEM image showing the morphology of the Cu_xSe_y film on FTO substrate upon attempt to exchange Zn^{2+} and Cd^{2+} simultaneously (in one pot) (b) SEM-EDS spectra showing only incorporation of Cd into the host film with neglectable Zn incorporation (c) UV-Vis absorbance spectra of the product film after exchange with respect to the pristine Cu_xSe_y film.

Sample	Cu μmol	Zn μmol	Cd μmol	Se μmol	Cu:Se
Cu_xSe_y-Zn-Cd-2hr	0.465	0.187	0.270	0.492	0.947
Cu_xSe_y-Zn-Cd-1hr	0.584	0.181	0.360	0.618	0.944
Cu_xSe_y-Zn-Cd-1hr-2	0.740	0.263	0.459	0.788	0.938
**Average**	**0.662**	**0.222**	**0.409**	**0.703**	**0.941**
St. Dev	0.078	0.041	0.050	0.085	0.003
St. Error	0.055	0.029	0.035	0.060	0.002

Table S18: ICP-MS analysis by digestion in nitric acid of three first-generation samples of the sequential cation exchange between Cu_xSe_y films and Zn^{2+},Cd^{2+} (Cu_xSe_y-Zn-Cd) used to make bar graph inset in Figure 2f.

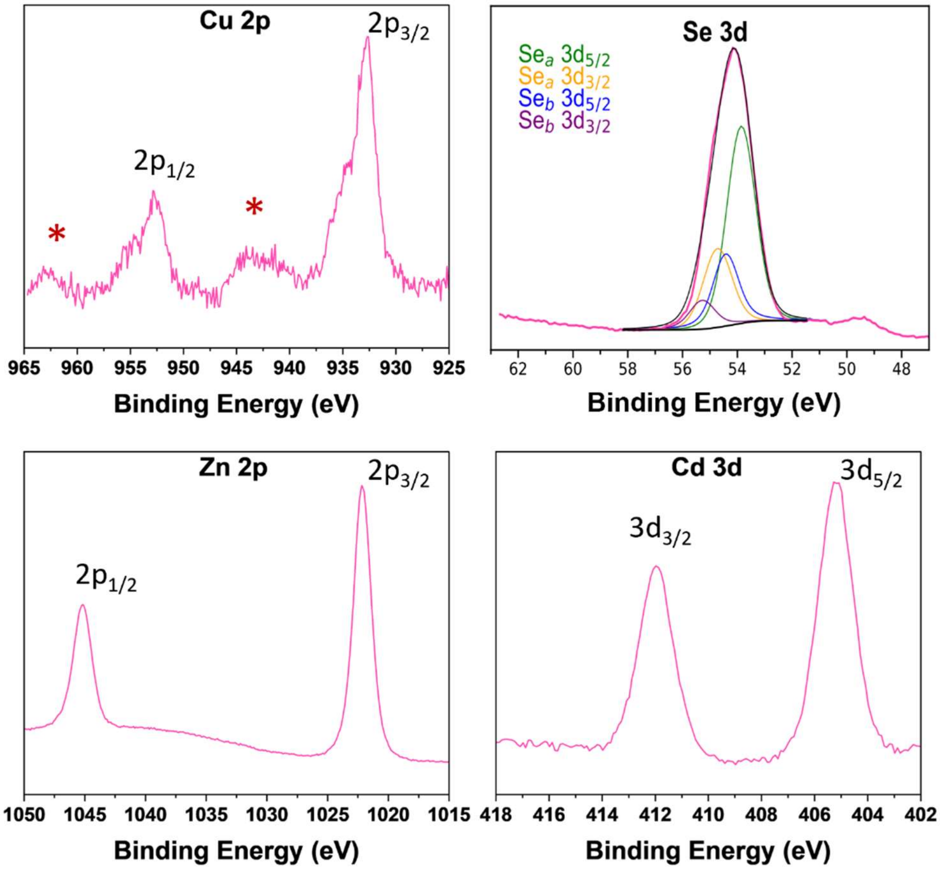

Figure S30: XPS surface analysis of a first-generation sample of the sequential exchange between Cu_xSe_y films and Zn^{2+},Cd^{2+} (Cu_xSe_y-Zn-Cd). The Cu 2p spectrum aligns with that expected for Cu^{+}/Cu^{2+} showing the satellite peaks expected for Cu^{2+}.The deconvoluted Se 3d spectrum shows the presence of a selenide with likely other selenium species. The Zn spectrum is consistent with Zn^{2+} based on a FWHM of value of 1.7 eV for the $2p_{3/2}$ peak.[12] Determination of Zn oxidation state based on the binding energy is difficult as there is great overlap.[3] The Cd $3d_{5/2}$ and $3d_{3/2}$ binding energies of 405.3 eV and 411.9 eV as expected for Cd^{2+}.[3,4]

Sample	Peak	Average Atomic Percentage	St. Dev
Cu_xSe_y-Zn-Cd	Cu 2p3	6.2	0.1
	Se 3d	51.5	1.1
	Zn 2p3	37.0	1.0
	Cd 3d	5.3	0.1

Table S19: XPS surface quantification of a first-generation sample of the sequential exchange between Cu_xSe_y films and Zn^{2+},Cd^{2+} (Cu_xSe_y-Zn-Cd) with the average atomic percentage calculated across three regions of the sample.

Rxn	Precursor salt	Solvent	[Ag] µmol	[Cd] µmol	Ag/Cd
8.1	TFA	Toluene	0.310	0.171	1.808
8.2	TFA	Toluene	0.351	0.178	1.970
8.3	TFA	Toluene	0.412	0.086	4.783
2_27_23	TFA	Toluene	0.240	0.080	3.019
**Average**	**TFA**	**Toluene**	**0.328**	**0.129**	**2.895**
St. Dev	TFA	Toluene	0.062	0.046	1.185
St. Error	TFA	Toluene	0.024	0.024	0.789
10.1	TFA	THF	0.269	0.104	2.577
10.2	TFA	THF	0.341	0.095	3.592
10.3	TFA	THF	0.302	0.073	4.158
2_27_23	TFA	THF	0.291	0.078	3.722
**Average**	**TFA**	**THF**	**0.301**	**0.088**	**3.512**
St. Dev	TFA	THF	0.026	0.013	0.579
St. Error	TFA	THF	0.013	0.006	0.290
11.1	TFA	ACN	0.269	0.187	1.440
11.2	TFA	ACN	0.275	0.146	1.879
11.3	TFA	ACN	0.291	0.199	1.461
2_27_23	TFA	ACN	0.256	0.140	1.828
**Average**	**TFA**	**ACN**	**0.273**	**0.168**	**1.652**
St. Dev	TFA	ACN	0.012	0.025	0.203
St. Error	TFA	ACN	0.006	0.013	0.101
12.1	TFA	EtOH	0.318	0.227	1.398
12.2	TFA	EtOH	0.313	0.120	2.607
12.3	TFA	EtOH	0.298	0.145	2.051
2_27_23	TFA	EtOH	0.233	0.125	1.863
**Average**	**TFA**	**EtOH**	**0.290**	**0.154**	**1.980**
St. Dev	TFA	EtOH	0.034	0.043	0.433
St. Error	TFA	EtOH	0.017	0.022	0.217
3.1	TFA	MeOH	0.230	0.114	2.011
3.2	TFA	MeOH	0.311	0.108	2.883
3.3	TFA	MeOH	0.216	0.060	3.592
2_27_23	TFA	MeOH	0.238	0.108	2.196
**Average**	**TFA**	**MeOH**	**0.248**	**0.098**	**2.671**
St. Dev	TFA	MeOH	0.037	0.022	0.624
St. Error	TFA	MeOH	0.018	0.011	0.312
13.1	TFA	H_2O	0.336	0.093	3.618
13.2	TFA	H_2O	0.284	0.080	3.552
13.3	TFA	H_2O	0.276	0.120	2.306
2_27_23	TFA	H_2O	0.290	0.118	2.454
**Average**	**TFA**	**H_2O**	**0.296**	**0.103**	**2.982**
St. Dev	TFA	H_2O	0.024	0.017	0.605
St. Error	TFA	H_2O	0.012	0.008	0.303

Table S20: ICP-MS analysis by digestion in nitric acid of the first-generation exchange between CdS films and Ag^+ used in the solvent study with silver trifluoroacetate (TFA) as precursor. All reactions were carried out for 1 hour at room temperature. The data herein was used to create the bar graphs and corresponding error bars shown in Figure 3d of the main text.

Rxn	Precursor salt	Solvent	[Ag] µmol	[Cd] µmol	Ag/Cd
1.1	TFB	MeOH	0.517	0.226	2.284
1.2	TFB	MeOH	0.348	0.193	1.805
1.3	TFB	MeOH	0.211	0.080	2.635
2_27_23	TFB	MeOH	0.276	0.052	5.300
**Average**	**TFB**	**MeOH**	**0.338**	**0.138**	**3.006**
St. Dev	TFB	MeOH	0.114	0.073	1.357
St. Error	TFB	MeOH	0.057	0.037	0.678
2.1	p-TS	MeOH	0.303	0.166	1.829
2.2	p-TS	MeOH	0.313	0.230	1.359
2.3	p-TS	MeOH	0.245	0.192	1.276
2_27_23	p-TS	MeOH	0.225	0.056	4.057
**Average**	**p-TS**	**MeOH**	**0.272**	**0.161**	**2.130**
St. Dev	p-TS	MeOH	0.037	0.065	1.132
St. Error	p-TS	MeOH	0.019	0.033	0.566
3.1	TFA	MeOH	0.230	0.114	2.011
3.2	TFA	MeOH	0.311	0.108	2.883
3.3	TFA	MeOH	0.216	0.060	3.592
2_27_23	TFA	MeOH	0.238	0.108	2.196
**Average**	**TFA**	**MeOH**	**0.248**	**0.094**	**2.829**
St. Dev	TFA	MeOH	0.037	0.022	0.624
St. Error	TFA	MeOH	0.018	0.011	0.312
4.1	triflate	MeOH	0.289	0.116	2.488
4.2	triflate	MeOH	0.327	0.192	1.703
4.3	triflate	MeOH	0.297	0.148	2.011
2_27_23	triflate	MeOH	0.201	0.047	4.318
**Average**	**triflate**	**MeOH**	**0.278**	**0.126**	**2.630**
St. Dev	triflate	MeOH	0.047	0.053	1.014
St. Error	triflate	MeOH	0.023	0.026	0.507
5.1	Nitrate	MeOH	0.282	0.216	1.305
5.2	Nitrate	MeOH	0.324	0.087	3.708
5.3	Nitrate	MeOH	0.217	0.060	3.638
2_27_23	Nitrate	MeOH	0.216	0.052	4.121
**Average**	**Nitrate**	**MeOH**	**0.260**	**0.104**	**3.193**
St. Dev	Nitrate	MeOH	0.046	0.066	1.106
St. Error	Nitrate	MeOH	0.023	0.033	0.553

Table S21: ICP-MS analysis by digestion in nitric acid of the first-generation exchange between CdS films and Ag^+ used in the precursor salt study with methanol as solvent. All reactions were carried out for 1 hour at room temperature. The data herein was used to create bar graphs and corresponding error bars shown in Figure 3e of the main text.

Rxn	Precursor salt	Solvent	[Ag] µmol	[Cd] µmol	Ag:Cd
6.1	Neodec	Toluene	0.056	0.283	0.199
6.2	Neodec	Toluene	0.051	0.366	0.140
6.3	Neodec	Toluene	0.093	0.370	0.250
2_27_23	Neodec	Toluene	0.026	0.135	0.193
**Average**	**Neodec**	**Toluene**	**0.057**	**0.289**	**0.196**
St. Dev	Neodec	Toluene	0.024	0.095	0.039
St. Error	Neodec	Toluene	0.012	0.048	0.019
7.1	TFB	Toluene	0.648	0.037	17.401
7.2	TFB	Toluene	0.409	0.032	12.852
7.3	TFB	Toluene	0.463	0.035	13.429
7.4	TFB	Toluene	0.609	0.054	11.331
2_27_23	TFB	Toluene	0.263	0.068	3.893
**Average**	**TFB**	**Toluene**	**0.479**	**0.045**	**11.781**
St. Dev	TFB	Toluene	0.139	0.014	4.424
St. Error	TFB	Toluene	0.062	0.006	1.979
8.1	TFA	Toluene	0.310	0.171	1.808
8.2	TFA	Toluene	0.351	0.178	1.970
8.3	TFA	Toluene	0.412	0.086	4.783
2_27_23	TFA	Toluene	0.240	0.080	3.019
**Average**	**TFA**	**Toluene**	**0.328**	**0.129**	**2.895**
St. Dev	TFA	Toluene	0.062	0.046	1.185
St. Error	TFA	Toluene	0.031	0.023	0.593
9.1	triflate	Toluene	0.307	0.115	2.671
9.2	triflate	Toluene	0.323	0.172	1.874
9.3	triflate	Toluene	0.243	0.095	2.562
2_27_23	triflate	Toluene	0.227	0.060	3.813
**Average**	**triflate**	**Toluene**	**0.275**	**0.110**	**2.730**
St. Dev	triflate	Toluene	0.041	0.041	0.696
St. Error	triflate	Toluene	0.020	0.020	0.348

Table S22: ICP-MS analysis by digestion in nitric acid of first-generation exchange between CdS films and Ag^{+} used in the precursor salt study with toluene as solvent. All reactions were carried out for 1 hour at room temperature except the reaction with silver neodecanoate which was ran for 3 hours at 65 °C. The data herein was used to create bar graphs and corresponding error bars shown in Figure 3f of the main text. (neodec = neodecanoate, TFB = tetrfluoroborate, TFA = trifluoroacetate).

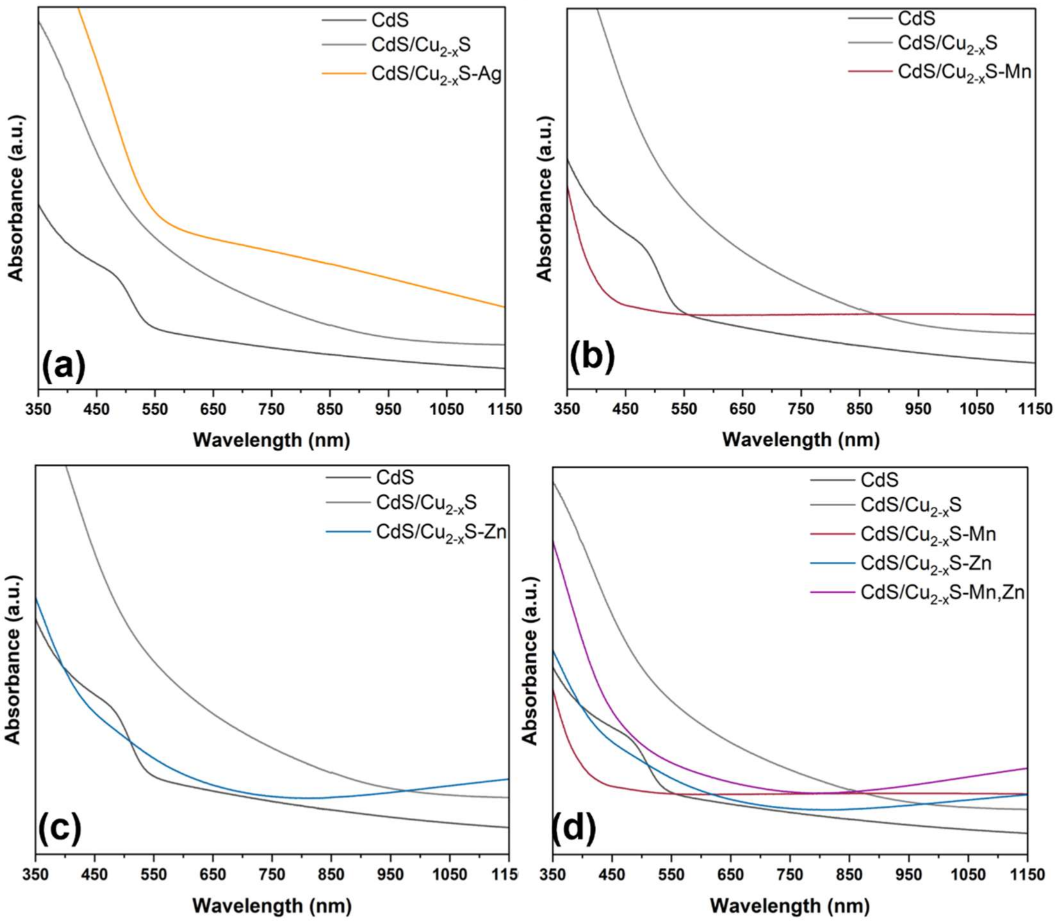

Figure S31: UV-Vis absorbance spectra of second-generation exchanges with respect to CdS/$Cu_{2-x}S$ and pristine CdS films with (a) Ag^+, (b) $Mn^{2+,}$ (c) Zn^{2+} and (d) simultaneously Mn^{2+} and Zn^{2+} in one pot (purple) in comparison with the individual CE reactions.

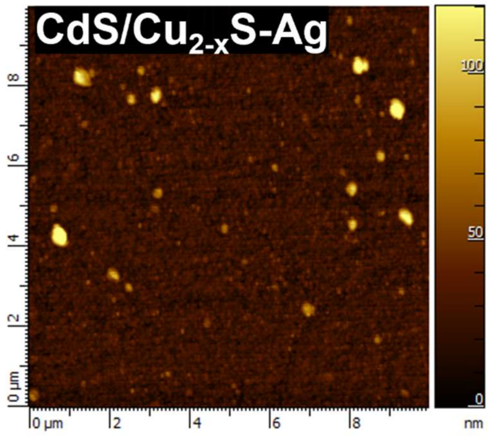

Figure S32: Two-dimensional AFM image obtained after the second-generation cation exchange reaction between $CdS/Cu_{2-x}S$ films and Ag^+ ($CdS/Cu_{2-x}S$-Ag). No noticeable differences were observed on the topography with an R_{ms} of 11.8 ± 0.6, close to the 10.3 ± 5.2 nm for $CdS/Cu_{2-x}S$ and 12.2 ± 1.1 nm for the pristine CdS films. See Table S1 for R_{ms} data.

Sample	[Cd] µmol	[Cu] µmol	[Ag] µmol	Ag:Cu
$CdS/Cu_{2-x}S$-Ag_1	0.013	0.016	0.365	22.815
$CdS/Cu_{2-x}S$-Ag_2	0.076	0.014	0.463	34.273
$CdS/Cu_{2-x}S$-Ag_3	0.012	0.023	0.175	7.760
Average	0.034	0.017	0.334	
Standard Deviation	0.036	0.005	0.146	
Standard Error	0.021	0.003	0.084	

Table S23: ICP-MS analysis by digestion in nitric acid of three second-generation samples of the exchange between of $CdS/Cu_{2-x}S$ films and Ag^+ ($CdS/Cu_{2-x}S$-Ag) used to create bar graph insets and corresponding error bars in Figure 5b of the main text.

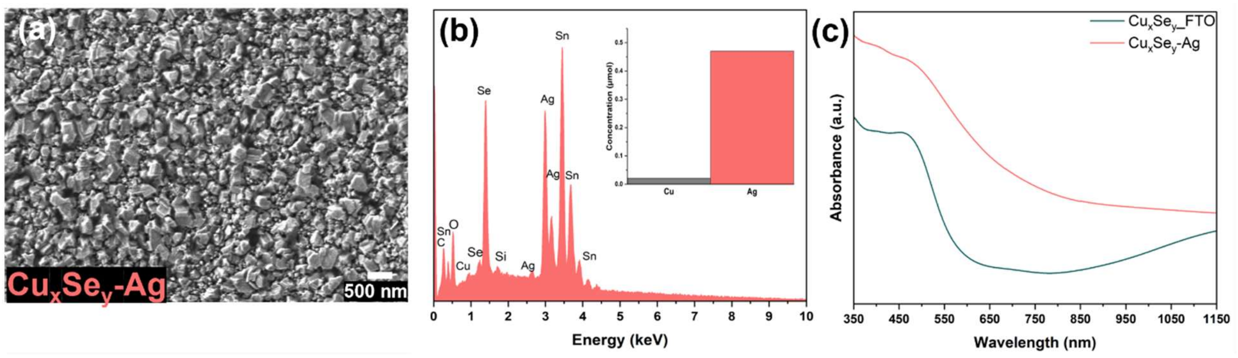

Figure S33: (a) SEM image showing the morphology of the Cu_xSe_y film on FTO substrate upon exchange with Ag^+. (b) SEM-EDS spectra with inset showing the cation concentrations obtained from ICP-MS analysis (c) UV-Vis absorbance spectra of the product film after exchange with respect to the pristine Cu_xSe_y film.

Sample	[Cd] µmol	[Cu] µmol	[Mn] µmol	Mn:Cu
$CdS/Cu_{2-x}S$-Mn_1	0.013	0.144	0.109	0.757
$CdS/Cu_{2-x}S$-Mn_2	0.007	0.235	0.103	0.436
$CdS/Cu_{2-x}S$-Mn_3	0.010	0.210	0.060	0.283
$CdS/Cu_{2-x}S$-Mn_4	0.007	0.071	0.202	2.861
$CdS/Cu_{2-x}S$-Mn_5	0.081	0.255	0.076	0.297
$CdS/Cu_{2-x}S$-Mn_6	0.023	0.076	0.180	2.379
$CdS/Cu_{2-x}S$-Mn_7	0.101	0.152	0.029	0.191
Average	0.035	0.163	0.108	
Standard Deviation	0.039	0.074	0.063	
Standard Error	0.015	0.028	0.024	

Table S24: ICP-MS analysis by digestion in nitric acid of seven samples of second-generation exchange between CdS/Cu_{2-x} S and Mn^{2+} ($CdS/Cu_{2-x}S$-Mn) used to create bar graph insets and corresponding error bars in Figure 5d of the main text.

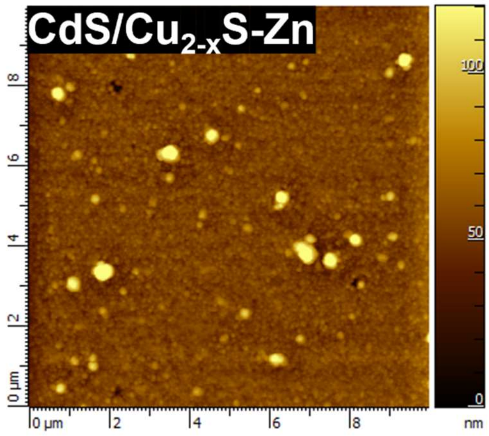

Figure S34: Two-dimensional AFM image obtained after the second-generation cation exchange reaction between $CdS/Cu_{2-x}S$ films and Zn^{2+} ($CdS/Cu_{2-x}S$-Zn). No noticeable differences were observed on the topography with an R_{ms} of 11.5 ± 2.3, close to the 10.3 ± 5.2 nm for $CdS/Cu_{2-x}S$ and 12.2 ± 1.1 nm for the pristine CdS films. See Table S2 for R_{ms} data.

Sample	[Cd] µmol	[Cu] µmol	[Zn] µmol	Zn:Cu
$CdS/Cu_{2-x}S$-Zn_1	0.025	0.148	0.089	0.601
$CdS/Cu_{2-x}S$-Zn_2	0.043	0.162	0.094	0.584
$CdS/Cu_{2-x}S$-Zn_3	0.017	0.110	0.090	0.818
Average	0.028	0.140	0.091	
Standard Deviation	0.013	0.027	0.003	
Standard Error	0.008	0.015	0.002	

Table S25: ICP-MS analysis by digestion in nitric acid of three samples of second-generation exchange between CdS/Cu_{2-x} S films and Zn^{2+} ($CdS/Cu_{2-x}S$-Zn) used to create bar graph insets and corresponding error bars in Figure 5f of the main text.

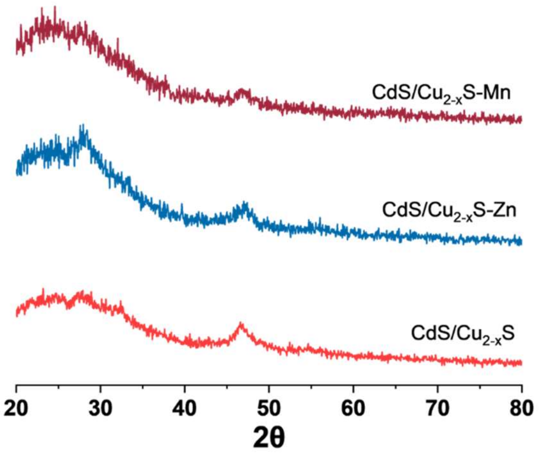

Figure S35: Grazing incidence XRD patterns of second-generation samples of the exchange between $CdS/Cu_{2-x}S$ films and Mn^{2+} ($CdS/Cu_{2-x}S$-Mn, top) and Zn^{2+} ($CdS/Cu_{2-x}S$-Zn, middle). The pattern for $CdS/Cu_{2-x}S$ (bottom) is included for comparison.

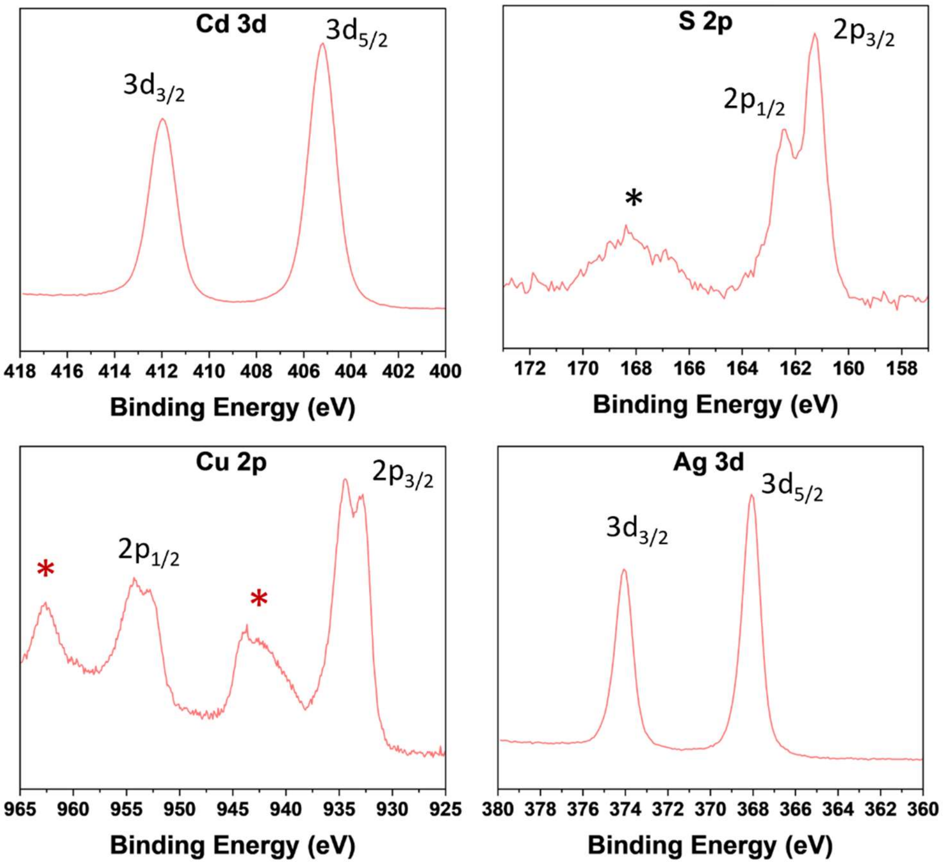

Figure S36: XPS surface analysis of a second-generation cation exchange reaction between CdS/$Cu_{2-x}S$ film and Ag^+ (CdS/$Cu_{2-x}S$-Ag). The Cu 2p spectrum aligns with that expected for Cu^+/Cu^{2+} showing the satellite peaks expected for Cu^{2+}. [3,5] The S 2p and Ag 3d spectra are consistent to that of other samples reported above. *Binding energy at 169 eV corresponding to sulfate species from surface oxidation.[3]

Sample	Peak	Average Atomic Percentage	St. Dev
CdS/$Cu_{2-x}S$-Ag	Cd 3d	38.0	9.8
	Cu 2p3	27.0	11.1
	Ag 3d	20.2	1.3
	S 2p	14.8	1.5

Table S26: XPS surface quantification of a second-generation cation exchange reaction between CdS/$Cu_{2-x}S$ films and Ag^+ (CdS/$Cu_{2-x}S$-Ag) average atomic percentage calculated across three regions of the sample.

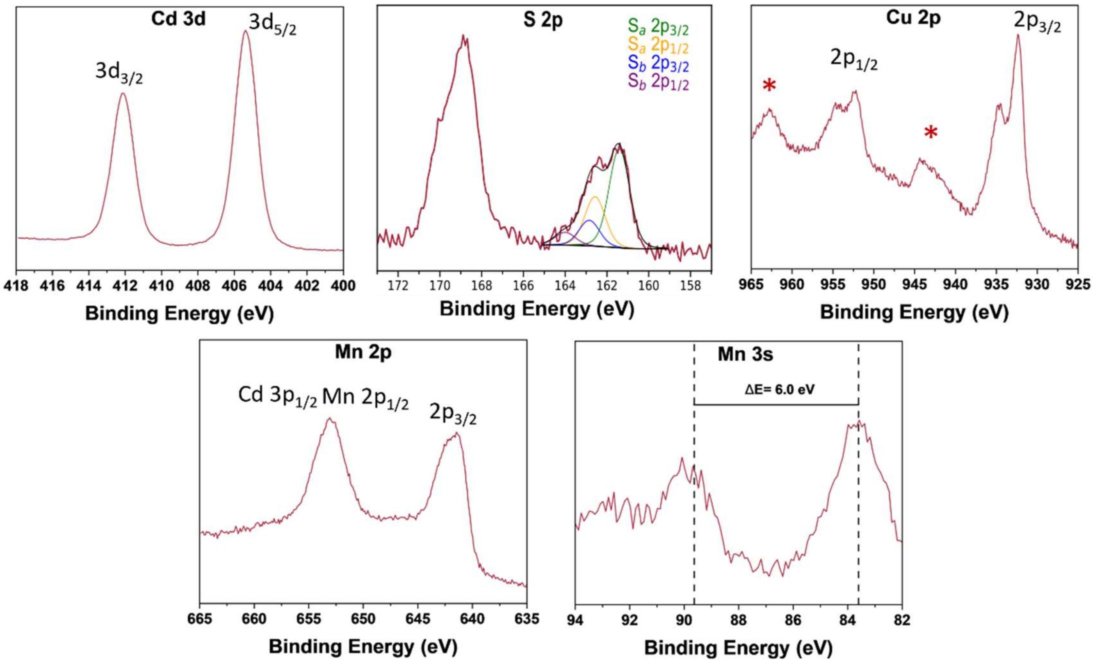

Figure S37: XPS surface analysis of a second-generation exchange between $CdS/Cu_{2-x}S$ and Mn^{2+} ($CdS/Cu_{2-x}S$-Mn). The Cu 2p spectrum aligns with that expected for Cu^{+}/Cu^{2+} showing the satellite peaks expected for Cu^{2+}. [3,5]The S 2 2p spectrum shows greater degree of oxidized species including the *binding energy at 169 eV corresponding to sulfate species. The Mn splitting of the Mn 3s spectrum is in agreement with Mn^{2+}.[3]

Sample	Peak	Average Atomic Percentage	St. Dev
$CdS/Cu_{2-x}S$-Mn	Cd 3d	34.5	2.8
	Cu 2p3	11.4	3.1
	Mn 2p3	33.0	3.2
	S 2p	21.1	2.7

Table S27: XPS surface quantification of a second-generation cation exchange reaction between $CdS/Cu_{2-x}S$ films and Mn^{2+} ($CdS/Cu_{2-x}S$-Mn) with the average atomic percentage calculated across three regions of the sample.

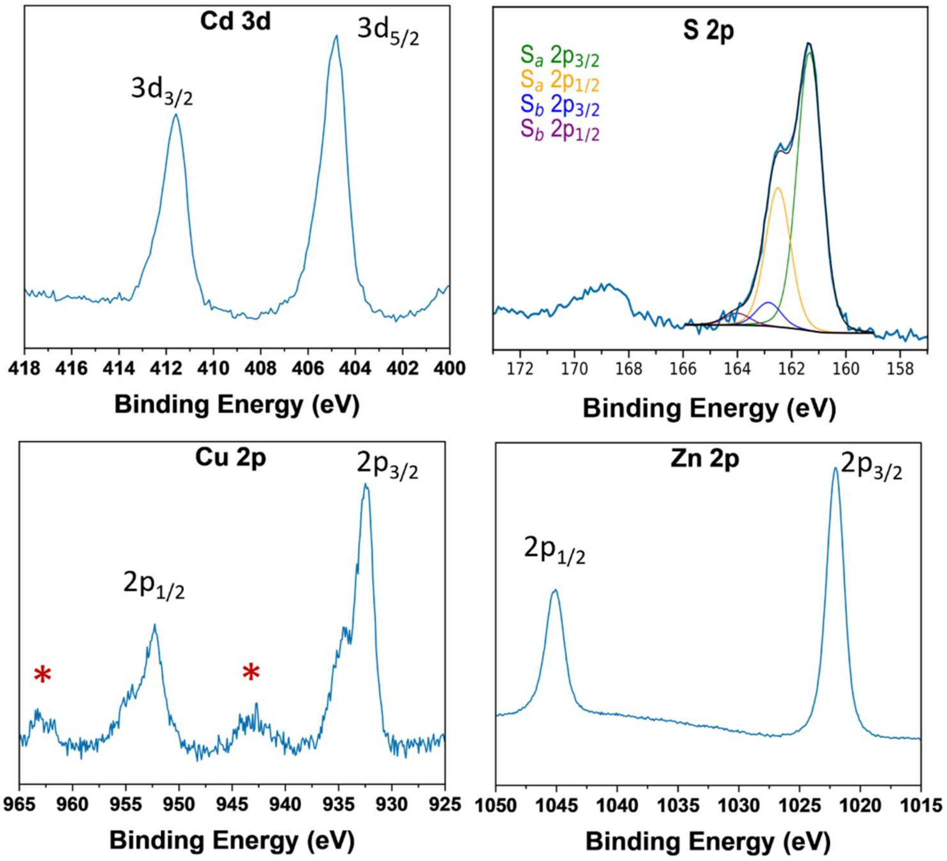

Figure S38: XPS surface analysis of second-generation exchange between CdS/Cu_{2-x} S and Zn^{2+} (CdS/Cu_{2-x}S-Zn). The Cu 2p spectrum aligns with that expected for Cu^{+}/Cu^{2+} showing the satellite peaks expected for Cu^{2+}. The S 2 2p spectrum shows greater degree of oxidized sulfides. The *binding energy at 169 eV corresponding to sulfate species. The Zn spectrum is consistent with Zn^{2+} based on a FWHM of value of 1.6 eV for the $2p_{3/2}$ peak. [12]

Sample	Peak	Average Atomic Percentage	St. Dev
CdS/Cu_{2-x}S-Zn	Cd 3d	5.5	0.2
	Cu 2p3	4.1	0.9
	Zn 2p3	56.3	3.3
	S 2p	34.1	3.6

Table S28: XPS surface quantification of a second-generation cation exchange reaction between CdS/Cu_{2-x}S films and Zn^{2+} (CdS/Cu_{2-x}S-Zn) with the average atomic percentage calculated across three regions of the sample.

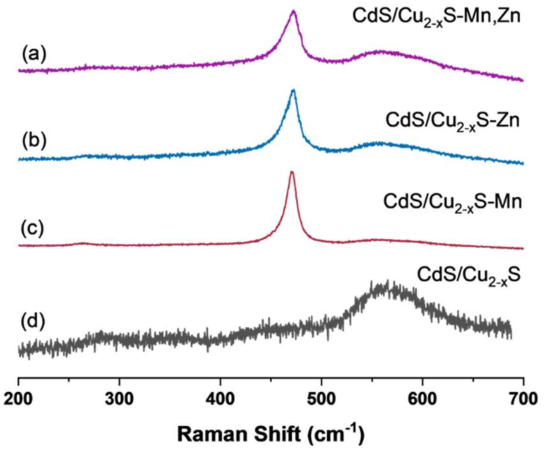

Figure S39: Raman spectra of second-generation samples where $CdS/Cu_{2-x}S$ films were exchanged with (a) Zn^{2+}, Mn^{2+} simultaneously ($CdS/Cu_{2-x}S$-ZnMn, top), (b) Zn^{2+} ($CdS/Cu_{2-x}S$-Zn), and (c) Mn^{2+} ($CdS/Cu_{2-x}S$-Mn). (d) Raman spectra for $CdS/Cu_{2-x}S$ film is included for comparison. The spectra for the second-generation samples show a peak around 470 cm^{-1} associated with S-S bonds that arise from surface oxidation.

Sample	[Cd] µmol	[Cu] µmol	[Mn] µmol	[Zn] µmol	(Mn+Zn): Cu
**$CdS/Cu_{2-x}S$-Mn,Zn_1**	0.014	0.200	0.038	0.027	0.328
**$CdS/Cu_{2-x}S$-Mn,Zn_2**	0.027	0.247	0.065	0.055	0.482
**$CdS/Cu_{2-x}S$-Mn,Zn_3**	0.014	0.166	0.050	0.114	0.992
**$CdS/Cu_{2-x}S$-Mn,Zn_4**	0.032	0.259	0.075	0.068	0.552
**$CdS/Cu_{2-x}S$-Mn,Zn_5**	0.019	0.179	0.041	0.032	0.404
**$CdS/Cu_{2-x}S$-Mn,Zn_6**	0.093	0.248	0.043	0.033	0.307
**Average**	0.033	0.217	0.052	0.055	
**Standard Deviation**	0.030	0.040	0.015	0.030	
**Standard Error**	0.012	0.016	0.006	0.012	

Table S29: ICP-MS analysis by digesting in nitric acid six samples of second-generation exchange between $CdS/Cu_{2-x}S$ films and Zn^{2+}, Mn^{2+} ($CdS/Cu_{2-x}S$-Mn,Zn) where both guest cations were added simultaneously in one-pot. This data was used to create the bar graph inset and corresponding error bars in Figure 5h of the main text.

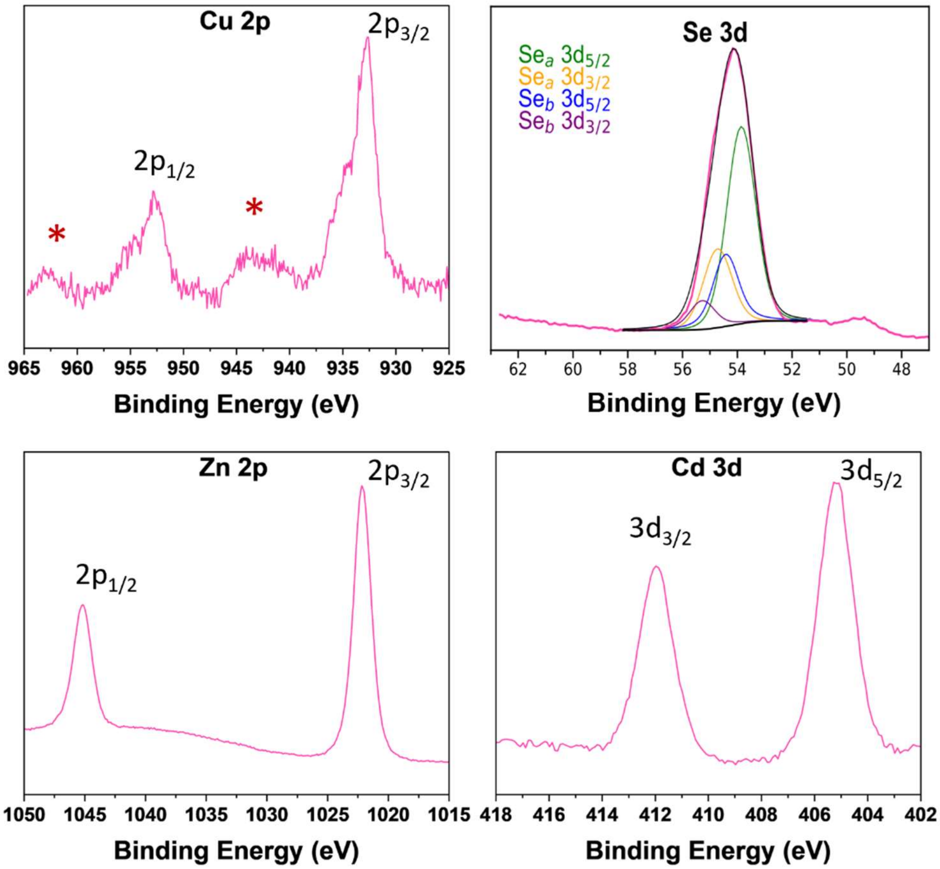

Figure S40: XPS surface analysis of second-generation exchange between CdS/$Cu_{2-x}S$ films and Zn^{2+}, Mn^{2+} (CdS/$Cu_{2-x}S$-ZnMn) where both guest cations were added simultaneously in one-pot.

Sample	Peak	Average Atomic Percentage	St. Dev	St. Error
CdS/$Cu_{2-x}S$-Mn,Zn	Cd 3d	5.2	0.9	0.2
	Cu 2p3	4.1	0.9	0.2
	Mn 2p3	56.3	1.8	0.0
	Zn 2p3	16.3	1.2	0.1
	S 2p	18.1	1.8	0.1

Table S30: XPS surface quantification of second-generation exchange between CdS/$Cu_{2-x}S$ films and Zn^{2+}, Mn^{2+}(CdS/$Cu_{2-x}S$-Mn,Zn) where both guest cations were added simultaneously in one-pot. The average atomic percentage calculated across three regions of the sample.

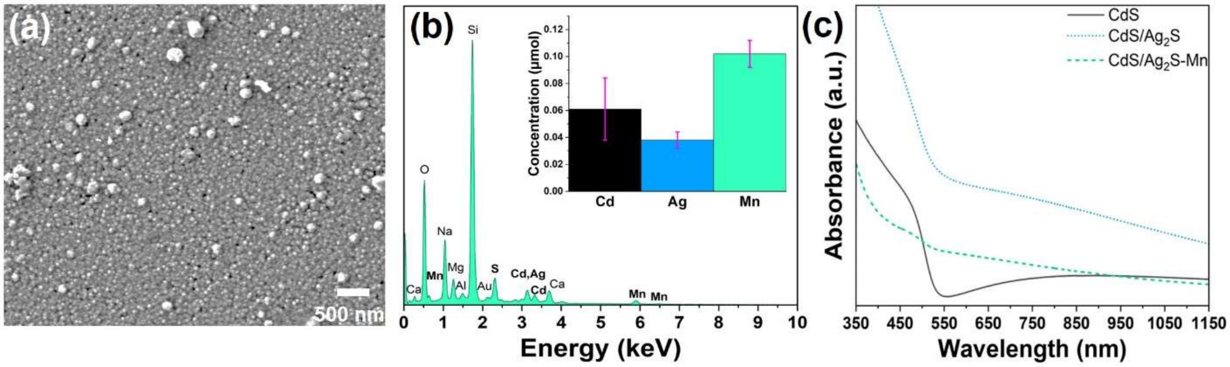

Figure S41: (a) SEM image showing the morphology of a CdS/Ag_2S film after exchange with Mn^{2+} (b) SEM-EDS spectrum with bar graph insets corresponding to the cation concentrations obtained from ICP-MS analysis of the films after exchange. The cation concentrations with error bars representative of the standard error of the mean were obtained from an averaging of three trials. (c) UV-Vis absorbance spectra of the product film after exchange with respect to CdS/Ag_2S and the pristine CdS film.

Sample	[Cd] µmol	[Ag] µmol	[Mn] µmol	Mn:Ag
CdS/Ag_2S-Mn_1	0.041	0.024	0.077	3.289
CdS/Ag_2S-Mn_2	0.116	0.048	0.118	2.482
CdS/Ag_2S-Mn_3	0.027	0.044	0.110	2.500
Average	0.061	0.038	0.102	
Standard Deviation	0.039	0.011	0.018	
Standard Error	0.023	0.006	0.010	

Table S31: ICP-MS analysis by digesting in nitric acid of three the second-generation exchange between CdS/Ag_2S films and Mn^{2+} (CdS/Ag_2S-Mn) used to create bar graph insets in Figure S41.

References

(1) Kumar, P.; Saxena, N.; Chandra, R.; Gupta, V.; Agarwal, A.; Kanjilal, D. Nanotwinning and Structural Phase Transition in CdS Quantum Dots. *Nanoscale Res. Lett.* **2012**, *7* (1), 584. https://doi.org/10.1186/1556-276X-7-584.

(2) Saleem, M. F.; Zhang, H.; Deng, Y.; Wang, D. Resonant Raman Scattering in Nanocrystalline Thin CdS Film. *J. Raman Spectrosc.* **2017**, *48* (2), 224–229. https://doi.org/10.1002/jrs.5002.

(3) *Handbook of X-Ray Photoelectron Spectroscopy: A Reference Book of Standard Spectra for Identification and Interpretation of XPS Data*, Update.; Moulder, J. F., Chastain, J., Eds.; Perkin-Elmer Corporation: Eden Prairie, Minn, 1992.

(4) Garza-Hernández, R.; Carrillo-Castillo, A.; Martínez-Landeros, V. H.; Martínez-Puente, M. A.; Martínez-Guerra, E.; Aguirre-Tostado, F. S. In-Situ X-Ray Photoelectron Spectroscopy Analysis of the Initial Growth of CdS Thin Films by Chemical Bath Deposition. *Thin Solid Films* **2019**, *682*, 142–146. https://doi.org/10.1016/j.tsf.2019.04.003.

(5) Cabrera-German, D.; García-Valenzuela, J. A.; Martínez-Gil, M.; Suárez-Campos, G.; Montiel-González, Z.; Sotelo-Lerma, M.; Cota-Leal, M. Assessing the Chemical State of Chemically Deposited Copper Sulfide: A Quantitative Analysis of the X-Ray Photoelectron Spectra of the Amorphous-to-Covellite Transition Phases. *Appl. Surf. Sci.* **2019**, *481*, 281–295. https://doi.org/10.1016/j.apsusc.2019.03.054.

(6) Baranov, A. V.; Rakovich, Yu. P.; Donegan, J. F.; Perova, T. S.; Moore, R. A.; Talapin, D. V.; Rogach, A. L.; Masumoto, Y.; Nabiev, I. Effect of ZnS Shell Thickness on the Phonon Spectra in CdSe Quantum Dots. *Phys. Rev. B* **2003**, *68* (16), 165306. https://doi.org/10.1103/PhysRevB.68.165306.

(7) Sharma, S. D.; Bayikadi, K.; Raman, S.; Neeleshwar, S. Structural, Morphological and Thermoelectric Properties of Self-Decorated Copper Selenide Nanosheets Synthesized at Room Temperature. *Curr. Appl. Phys.* **2022**, *40*, 74–82. https://doi.org/10.1016/j.cap.2020.06.010.

(8) Arulraj, A.; Ilayaraja, N.; Rajeshkumar, V.; Ramesh, M. Direct Synthesis of Cubic Shaped Ag2S on Ni Mesh as Binder-Free Electrodes for Energy Storage Applications. *Sci. Rep.* **2019**, *9* (1), 10108. https://doi.org/10.1038/s41598-019-46583-0.

(9) Sadanaga, R.; Sueno, S. X-RAY STUDY ON THE α-β TRANSITION OF Ag2S. *Mineral. J.* **1967**, *5* (2), 124–143. https://doi.org/10.2465/minerj1953.5.124.

(10) Hernández-Pagán, E. A.; O'Hara, A.; Arrowood, S. L.; McBride, J. R.; Rhodes, J. M.; Pantelides, S. T.; Macdonald, J. E. Transformation of the Anion Sublattice in the Cation-Exchange Synthesis of Au2S from Cu2–xS Nanocrystals. *Chem. Mater.* **2018**, *30* (24), 8843–8851. https://doi.org/10.1021/acs.chemmater.8b03814.

(11) Parker, G. K.; Watling, K. M.; Hope, G. A.; Woods, R. A SERS Spectroelectrochemical Investigation of the Interaction of Sulfide Species with Gold Surfaces. *Colloids Surf. Physicochem. Eng. Asp.* **2008**, *318* (1), 151–159. https://doi.org/10.1016/j.colsurfa.2007.12.029.

(12) Biesinger, M.C. *X-ray Photoelectron Spectroscopy (XPS) Reference Pages*. Zinc. http://www.xpsfitting.com/search/label/Zinc (accessed 2024-10-21).
